# Heme oxygenase-1 is an equid alphaherpesvirus 8 replication restriction host protein and suppresses viral replication via the PKCβ/ERK1/ERK2 and NO/cGMP/PKG pathway

**DOI:** 10.1128/spectrum.03220-23

**Published:** 2024-03-05

**Authors:** Tongtong Wang, Shuwen Li, Xinyao Hu, Yiqing Geng, Li Chen, Wenqiang Liu, Juan Zhao, Wenxia Tian, Changfa Wang, Yubao Li, Liangliang Li

**Affiliations:** 1 College of Agronomy, Liaocheng University, Liaocheng, Shandong, China; 2 College of Veterinary Medicine, Shanxi Agricultural University, Taigu, Shanxi, China; Oklahoma State University College of Veterinary Medicine, Stillwater, Oklahoma, USA

**Keywords:** EqHV-8, HO-1, biliverdin, anti-viral effect, mouse model

## Abstract

Equid alphaherpesvirus 8 (EqHV-8) is one of the most economically important viruses that is known to cause severe respiratory disease, abortion, and neurological syndromes in equines. However, no effective vaccines or therapeutic agents are available to control EqHV-8 infection. Heme oxygenase-1 (HO-1) is an antioxidant defense enzyme that displays significant cytoprotective effects against different viral infections. However, the literature on the function of HO-1 during EqHV-8 infection is little. We explored the effects of HO-1 on EqHV-8 infection and revealed its potential mechanisms. Our results demonstrated that HO-1 induced by cobalt-protoporphyrin (CoPP) or HO-1 overexpression inhibited EqHV-8 replication in susceptible cells. In contrast, HO-1 inhibitor (zinc protoporphyria) or siRNA targeting HO-1 reversed the anti-EqHV-8 activity. Furthermore, biliverdin, a metabolic product of HO-1, mediated the anti-EqHV-8 effect of HO-1 via both the protein kinase C (PKC)β/extracellular signal-regulated kinase (ERK)1/ERK2 and nitric oxide (NO)-dependent cyclic guanosine monophosphate (cGMP)–protein kinase G (PKG) signaling pathways. In addition, CoPP protected the mice by reducing the EqHV-8 infection in the lungs. Altogether, these results indicated that HO-1 can be developed as a promising therapeutic strategy to control EqHV-8 infection.

**IMPORTANCE:**

EqHV-8 infections have threatened continuously donkey and horse industry worldwide, which induces huge economic losses every year. However, no effective vaccination strategies or drug against EqHV-8 infection until now. Our present study found that one host protien HO-1 restrict EqHV-8 replication *in vitro* and *in vivo*. Furthermore, we demonstrate that HO-1 and its metabolite biliverdin suppress EqHV-8 relication via the PKCβ/ERK1/ERK2 and NO/cGMP/PKG pathways. Hence, we believe that HO-1 can be developed as a promising therapeutic strategy to control EqHV-8 infection.

## INTRODUCTION

Equid alphaherpesvirus 8 (EqHV-8) is known to cause severe respiratory diseases, abortion, and neurological disorders in equine, resulting in huge economic loss to the horse and donkey industry worldwide (1–3). However, no effective vaccines or specific anti-viral drugs are available to control EqHV-8 infections (4). Therefore, it is urgently required to develop efficient anti-viral strategies for combating EqHV-8 infections.

Heme oxygenase-1 (HO-1) was termed heat shock protein 32 and is encoded by the *HMOX1* gene (5). HO-1 serves as a cytoprotective enzyme that is induced in response to cellular stresses and inflammatory response (6, 7), and it is commonly expressed in mammalian cells. Numerous studies have reported that HO-1 is involved in antioxidant, anti-inflammatory, anti-apoptotic, and anti-microbial actions (8, 9). Recently, a study reported that HO-1 displays anti-viral activity against human influenza virus (10), human immunodeficiency virus (HIV) (11), human respiratory syncytial virus (12), dengue virus (DENV) (13), and hepatitis B virus infection (14). Similarly, HO-1 is known to suppress other viruses from animals, including porcine reproductive and respiratory syndrome virus (15), bovine viral diarrhea virus (BVDV) (16), pseudorabies virus (PRV) (17), and porcine circovirus type 3 (PCV3) (18). These studies have confirmed that HO-1 displays a broad-spectrum anti-viral activity. However, the role of HO-1 protein in EqHV-8 replication remains unclear.

It has been reported previously that biliverdin (BV), carbon monoxide (CO), and iron, the downstream metabolites of HO-1, participate in virus replication regulation and display anti-viral and anti-inflammatory activities (19, 20). BV is reduced to bilirubin (BR) by biliverdin reductase (BVR), which plays a crucial role in inhibiting lipid and protein peroxidation by clearing oxidative stress (21–23). For example, Lehmann et al. demonstrated that BV displays anti-HCV activity by increasing the anti-viral interferon response *in vitro* (24). A similar anti-viral effect of BV was observed in several viruses, such as human herpes simplex type 1 virus, enterovirus type 71, DENV, and HIV (25, 26). In addition, BR was found to exhibit anti-viral activity against porcine reproductive and respiratory syndrome virus (PRRSV, PCV3, and PRV) (17, 18, 27). CO is known to participate in different physiological activities and pathological processes, such as anti-inflammatory, anti-apoptotic effects, and to regulate cell proliferation (28). A previous study reported that CO suppresses BVDV replication *in vitro* (29).

Iron is a component of prosthetic groups in different enzymes and electron transfer proteins involved in redox reactions (30, 31). A recent study demonstrated that iron exerts anti-viral effects; for example, iron can suppress the expression of viral RNA and proteins by disturbing the enzymatic activity of the NS5B RNA polymerase (32, 33). However, the anti-viral effects of HO-1 against EqHV-8 infection and the underlying potential mechanisms remain unknown.

Here we investigated the function of HO-1 during EqHV-8 infection. The results indicated that HO-1 expression was significantly upregulated during EqHV-8 infection, and its induction or overexpression effectively inhibited EqHV-8 infection, which was reversed by siHO-1 or zinc protoporphyria (ZnPP) treatment. In addition, the anti-EqHV-8 activity of HO-1 metabolic products (BV, CO, and free iron) was explored. Our results showed that BV and secondary metabolite BR suppressed EqHV-8 replication via reactive oxygen species (ROS) reduction *in vitro* by activating the protein kinase C (PKC)β/extracellular signal-regulated kinase (ERK)1/ERK2 and the nitric oxide (NO)-dependent cyclic guanosine monophosphate (cGMP)/protein kinase G (PKG) pathways. The lung injury of cobalt-protoporphyrin (CoPP)-treated mice was significantly reduced. These data suggested that HO-1 and its metabolites, BV/BR, are involved in EqHV-8 replication *in vitro*, which could serve as a potential therapeutic agent for controlling EqHV-8 infections.

## RESULTS

### EqHV-8 infection decreases HO-1 expression in susceptible cells

**Fig 1:**
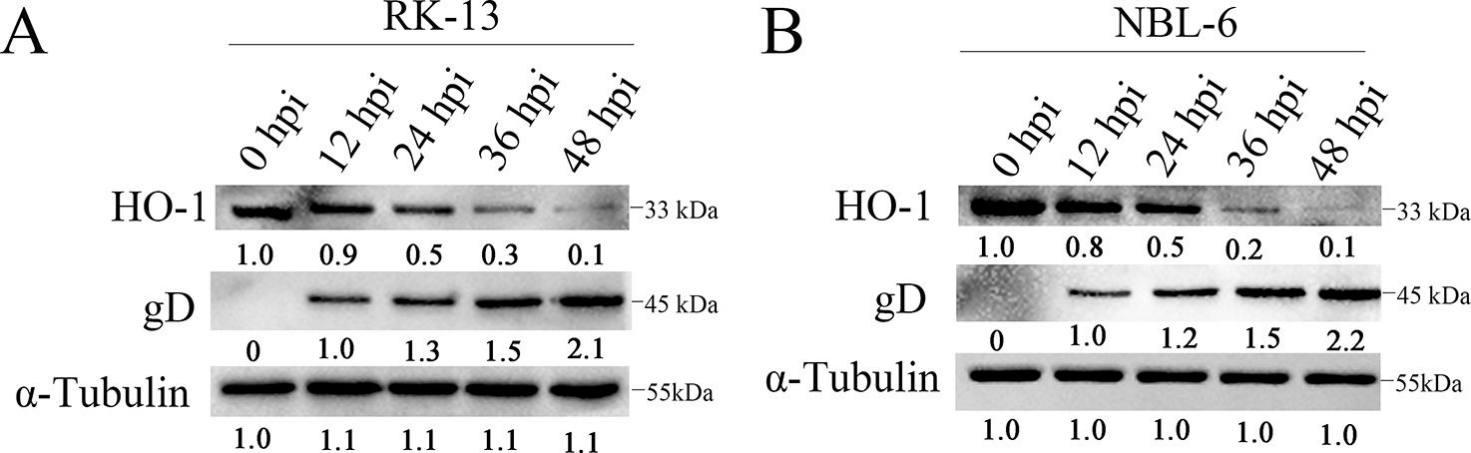
EqHV-8 infection reduces HO-1 expression in susceptible cells. RK-13 (**A**) and NBL-6 (**B**) cells were infected with EqHV-8 SDLC66 at an MOI of 0.1 and cultured at 37°C. Cell samples were collected at 0, 12, 24, 36, and 48 hpi. The expression of HO-1 was detected by Western blotting. α-Tubulin served as the loading control for Western blotting.

To assess HO-1 expression during the EqHV-8 infection, rabbit kidney 13 (RK-13) and NBL-6 cells infected with EqHV-8 [multiplicity of infection (MOI) of 0.1] were harvested at different times and analyzed by Western blotting. The expression of HO-1 significantly reduced at protein levels during the EqHV-8 infection process from 12, 24, 36, and 48 h post-infection (hpi) compared with the 0-hpi group in RK-13 cells in a time-dependent manner (Fig. 1A). Similar results were observed in NBL-6 cells (Fig. 1B).

### Upregulation of HO-1 activity suppresses EqHV-8 replication *in vitro*

**Fig 2:**
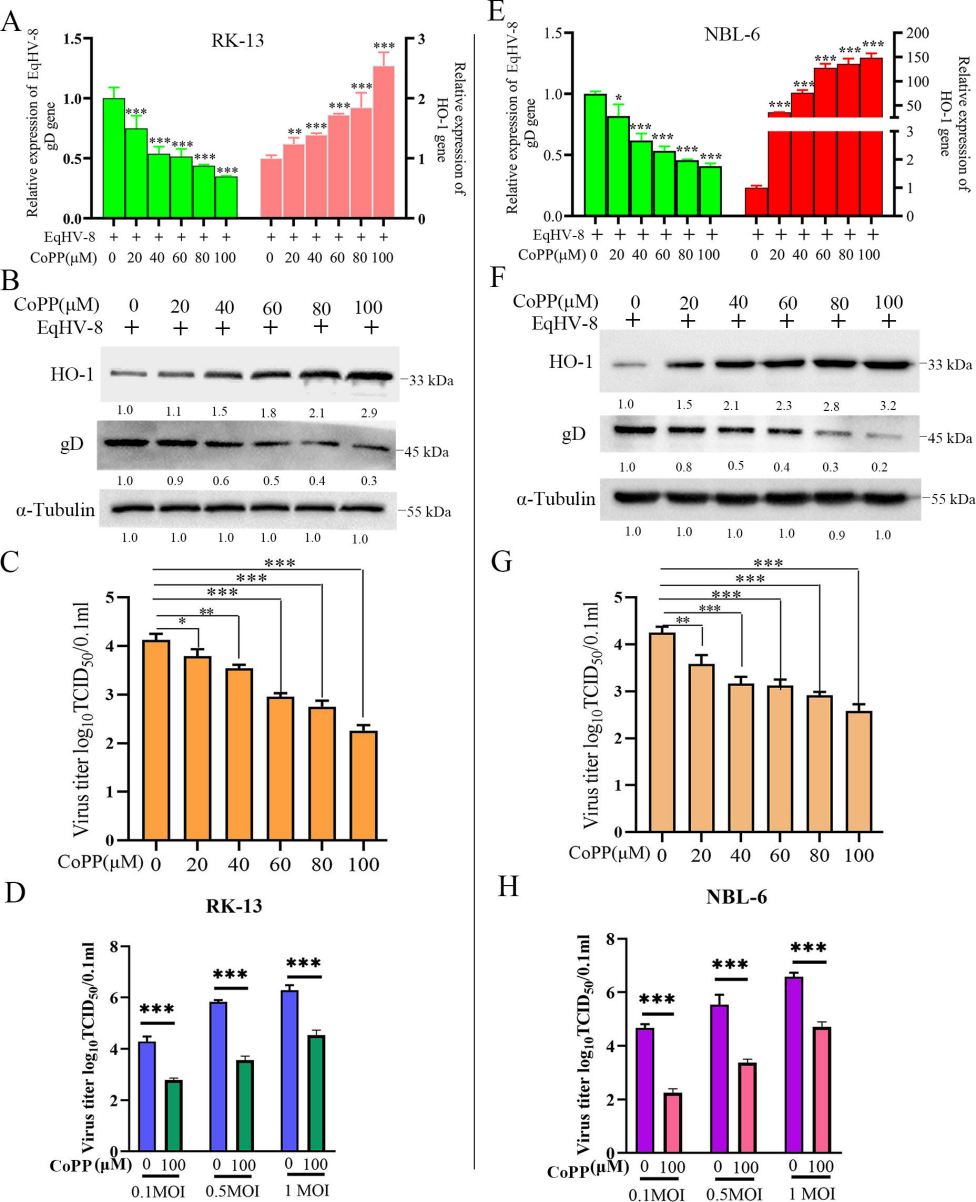
CoPP decreases EqHV-8 infection in susceptible cells by HO-1 induction. These susceptible cells were incubated in the presence or absence of different concentrations of CoPP for 12 h, followed by incubation with EqHV-8 SDLC66 at an MOI of 0.1. Subsequently, the cells were collected to analyze the expression of HO-1 and gD mRNAs and proteins at 24 hpi by qPCR and Western blotting, respectively, in RK-13 cells (**A and B**) and NBL-6 (**E and F**) cells. *GAPDH* served as the reference gene for qPCR, and α-tubulin served as the loading control for Western blotting. The cellular supernatants were harvested to determine the progeny virus titer in RK-13 (**C**) and NBL-6 (**G**) cells. RK-13 (**D**) and NBL-6 (**H**) were pre-treated with 0- or 100-µM CoPP, then inoculated with EqHV-8 at an MOI of 0.1, 0.5, and 1.0 for 24 h, respectively. The cellular supernatants were harvested to determine the progeny virus titer by TCID_50_. These data are presented as the mean ± SD of three independent experiments. *P < 0.05, **P < 0.01, ***P < 0.001. GAPDH, glyceraldehyde dehydrogenase.

To investigate whether the HO-1 enzyme activity was necessary for its anti-viral function, CoPP-induced HO-1 activity following EqHV-8 infection was determined in RK-13 and NBL-6 cells. First, the cytotoxicity of CoPP was assessed at different concentrations in these cells. The results demonstrated that RK-13 and NBL-6 cells treated with 100-µM CoPP at maximum had no significant cytotoxic effect compared with the untreated cells (Fig. S1). The HO-1 expression induced by CoPP and EqHV-8 replication was measured at 24 hpi in these cells by quantitative PCR (qPCR) and Western blotting. As expected, the results demonstrated that CoPP increased HO-1 expression both at mRNA and protein levels in a concentration-dependent manner in RK-13 cells (Fig. 2A), with a corresponding decrease in glycoprotein D (gD) expression (Fig. 2B). In addition, the cellular supernatant from these cells was collected to analyze the virus titer by 50% tissue culture infected dose (TCID_50_). Our data showed that the titer of EqHV-8 on RK-13 cells treated with different concentrations of CoPP significantly decreased in a concentration-dependent manner, compared with the control RK-13 cells (Fig. 2C). To evaluate if higher MOI can overcome the protective mechanism of CoPP, RK-13 cells were pre-treated with CoPP (100 µM) and inoculated at different MOIs (0.1, 0.5, and 1.0) of EqHV-8; the results showed that the virus titer by TCID_50_ in 100-µM CoPP-treated cells were significantly decreased compared with 0-µM CoPP-treated cells (Fig. 2D). Similar results were obtained in NBL-6 cells (Fig. 2E through H). These results demonstrated that CoPP treatment significantly reduced EqHV-8 replication in susceptible cells in a concentration-dependent manner.

### Decreased HO-1 enzyme activity reverses EqHV-8 replication

**Fig 3:**
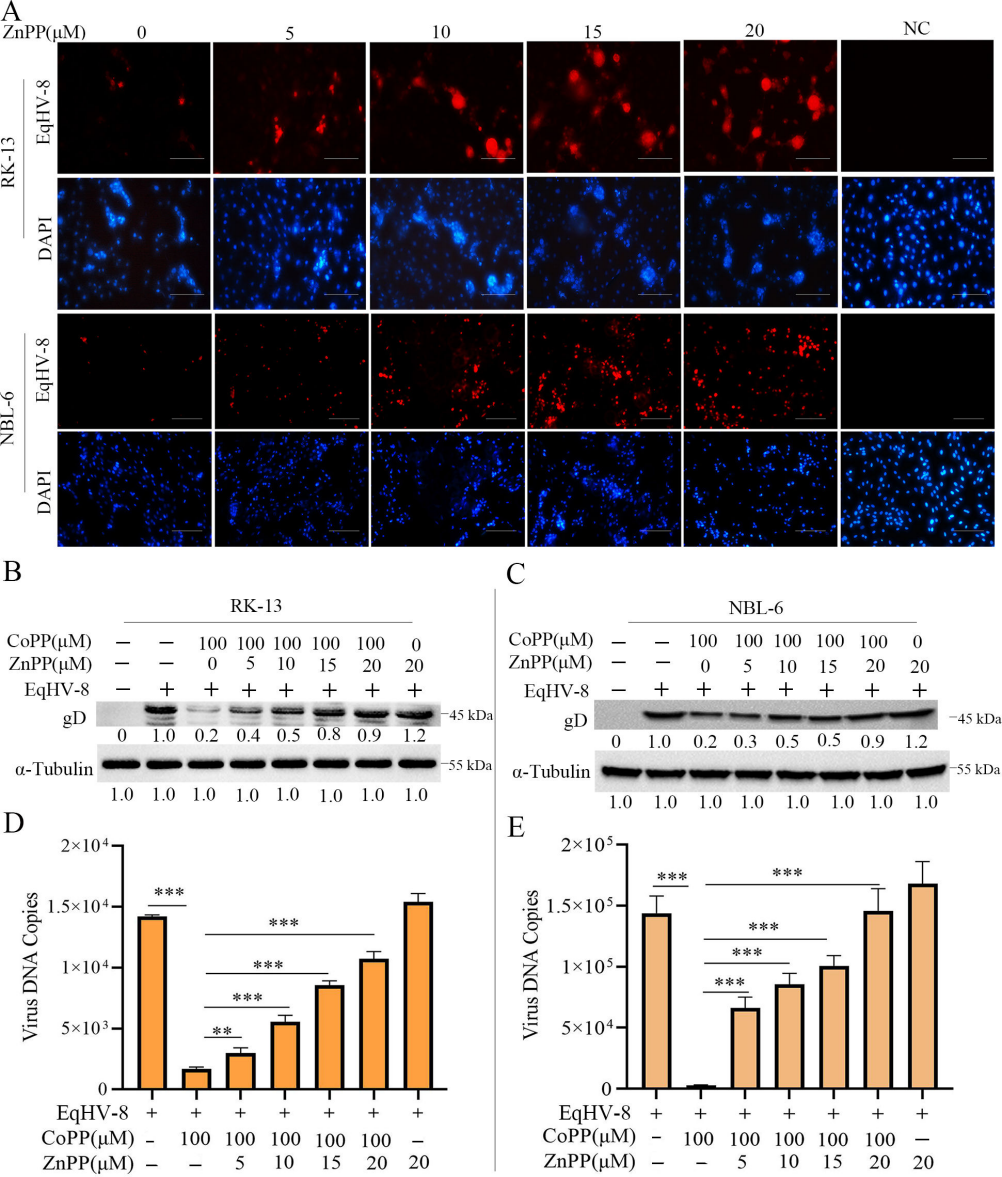
ZnPP reverses CoPP-induced anti-EqHV-8 effect. The RK-13 or NBL-6 cells were pre-treated with ZnPP at concentrations of 0, 5, 10, 15, and 20 µM for 12 h before EqHV-8 SDLC66 (MOI of 0.1) infection. The cells were fixed with 75% cold ethanol at 36 hpi, followed by IFA with the indicated antibody. Images were captured using Leica microsystem. Scale bar, 100 µm (**A**). EqHV-8-susceptible cells were pre-treated with ZnPP at different concentrations for 12 h, infected with EqHV-8 SDLC66 at an MOI of 0.1, followed by incubation with CoPP (100 µM) for 24 h. The gD protein expression was analyzed by Western blotting in RK-13 (**B**) and NBL-6 cells (**C**). The copy number of progeny virus was detected by qPCR in RK-13 (**D**) and NBL-6 cells (**E**). Data are presented as the mean ± SD of three independent experiments. **P < 0.01, ***P < 0.001. DAPI, 4′,6-diamidino-2-phenylindole.

ZnPP was used to further assess whether the activity of HO-1 was essential for its anti-EqHV-8 infection. First, the cytotoxicity of ZnPP was detected in these cells with cell counting kit-8 (CCK-8), and the results demonstrated that ZnPP at a maximum concentration of 20 µM exerted no significant effect on RK-13 or NBL-6 cells (Fig. S2). We next investigated whether ZnPP enhanced EqHV-8 replication *in vitro*. All cells were pre-treated with ZnPP at 5, 10, 15, and 20 µM for 12 h and were infected with EqHV-8 SDLC66 at an MOI of 0.1. Immunofluorescence assay (IFA) was performed at 24 hpi with mouse anti-EqHV-8-positive serum, and the results showed significantly increased EqHV-8 infection efficiency by ZnPP treatment (Fig. 3A). Subsequently, these cells were pre-treated with ZnPP at different concentrations for 12 h, infected with EqHV-8 SDLC66 at 0.1 MOI, followed by incubation with CoPP (100 µM) for 24 h. The cells and cellular supernatant were collected to analyze the expression of gD and progeny virus copies. As expected, CoPP-induced reduced expression of gD reversed with ZnPP treatment in RK-13 cells (Fig. 3B) and NBL-6 cells (Fig. 3C). A similar trend was observed in progeny virus copy number in RK-13 (Fig. 3D) and NBL-6 cells (Fig. 3E). In summary, these data indicated that HO-1 enzyme activity is essential for anti-EqHV-8 effect of endogenous HO-1.

### Overexpression of HO-1 inhibits EqHV-8 replication

**Fig 4:**
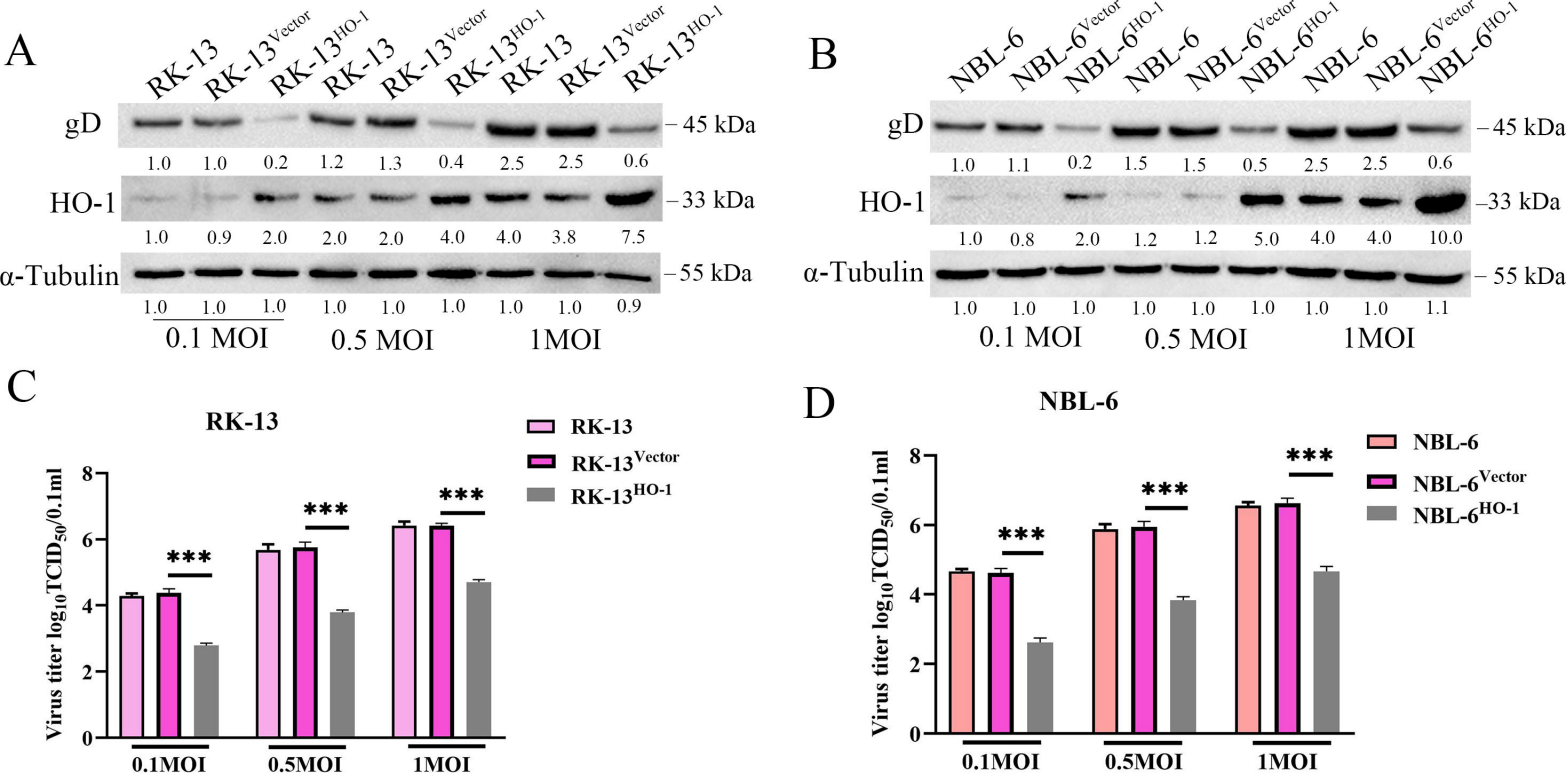
PiggyBac-mediated HO-1 overexpression suppresses EqHV-8 infection in RK-13 and NBL-6 cells. (**A and B**) RK-13^Vector^, RK-13^HO-1^ or NBL-6^Vector^, NBL-6^HO-1^ cells, and their parent cells were infected with EqHV-8 SDLC66 at MOIs of 0.1, 0.5, and 1.0, followed by collecting the cells at 24 hpi, and were subjected to Western blotting using the anti-gD antibody, anti-HO-1 antibody, or anti-α-tubulin antibody. The production of progeny viruses was measured by TCID_50_ in RK-13, RK-13^Vector^, RK-13^HO-1^ (**C**) or NBL-6, NBL-6^Vector^, and NBL-6^HO-1^ (**D**) cells. Data are presented as the mean ± SD of three independent experiments. ***P < 0.001.

HO-1 was overexpressed in RK-13 and NBL-6 cells using the piggyBac system and was named RK-13^HO-1^ and NBL-6^HO-1^. The overexpression was confirmed by Western blotting (Fig. S3). To further confirm EqHV-8 replication in these cells, new recombinant cells and parent cells (RK-13 and NBL-6 cells) were infected with EqHV-8 SDLC66 (MOIs of 0.1, 0.5, and 1.0) for 24 h. Furthermore, these cells and cellular supernatant were analyzed by Western blotting and TCID_50_. Compared with the vector-transfected cells, the gD protein expression was significantly decreased in RK-13^HO-1^ (Fig. 4A) and NBL-6^HO-1^ (Fig. 4B). In addition, the progeny virus titers in RK-13^HO-1^ were lower than those in RK-13^vector^ (Fig. 4C). The titers in NBL-6^HO-1^cells were also lower than those in NBL-6^vector^ (Fig. 4D). No difference was observed between RK-13^vector^, NBL-6^vector^, and their parent cells.

### HO-1 knockdown promotes EqHV-8 replication

**Fig 5:**
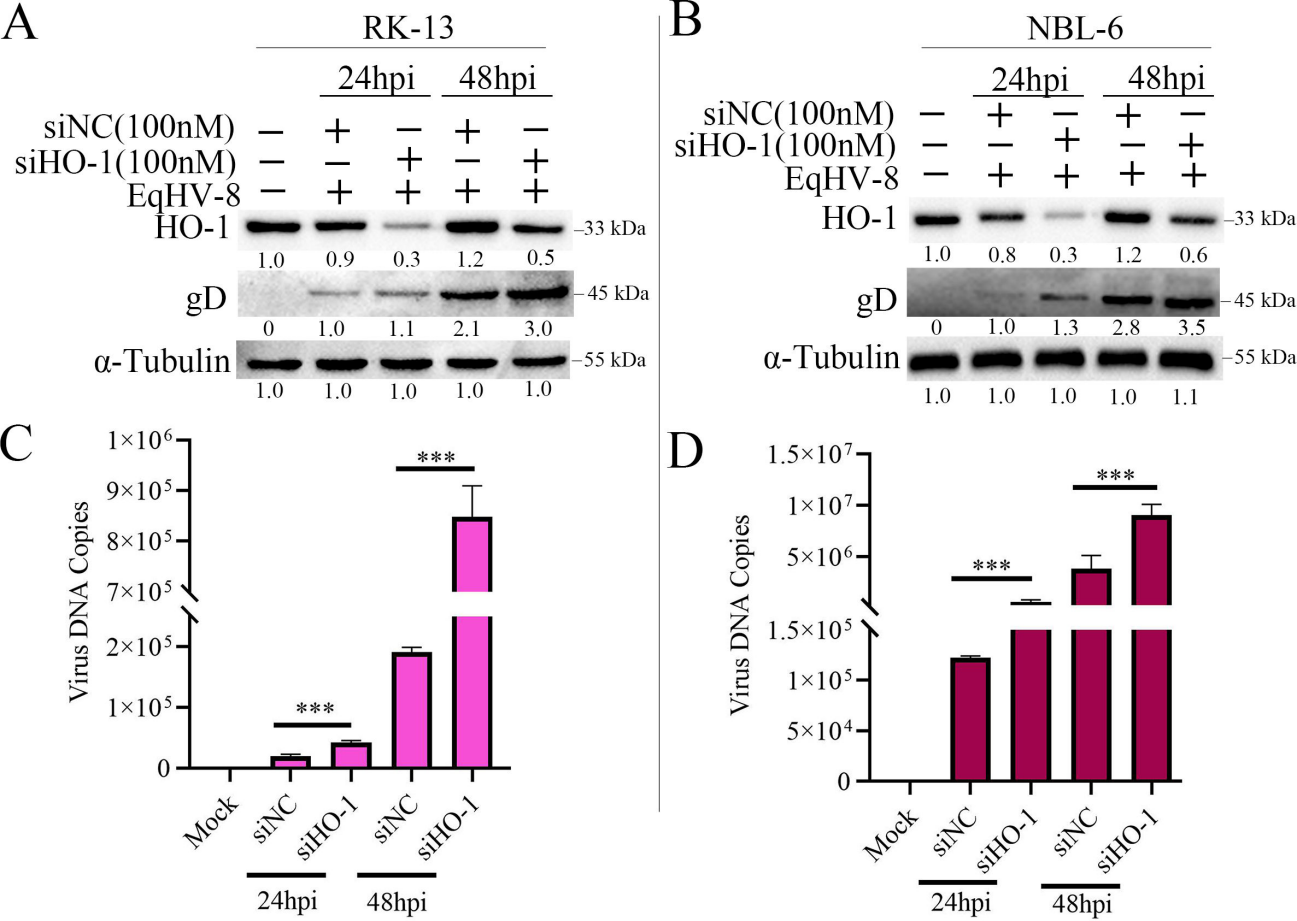
Knockdown endogenous HO-1 enhances EqHV-8 replication in RK-13 and NBL-6 cells. RK-13 (**A**) or NBL-6 (**B**) cells were transfected with siHO-1 or siNC for 12 h, followed by infection with SDLC66 at an MOI of 0.1. The cells and cellular supernatants were harvested to analyze the expressions of HO-1 and gD at the protein level by Western blotting at 24 and 48 hpi. The number of virus copies in the supernatant was detected via qPCR in RK-13 (**C**) and NBL-6 cells (**D**). Data are presented as the mean ± SD of three independent experiments. ***P < 0.001.

To further verify the effect of HO-1 on EqHV-8 replication, siHO-1 or siNC was transfected into RK-13 and NBL-6 cells for 48 h. The expression of HO-1 was measured by qPCR and Western blotting. siHO-1 reduced HO-1 expression significantly both at transcription and translation levels (Fig. S4). Subsequently, EqHV-8 replication of all cells with HO-1 knockdown was detected at 24 hpi via Western blotting and qPCR. The results revealed that gD expression in the siHO-1-transfected group was higher than in the siNC-treated group in RK-13 cells (Fig. 5A) and NBL-6 (Fig. 5B) cells. In addition, the copy number of progeny virus in the HO-1 knockdown group cells was higher than that in the siNC-transfected RK-13 cells (Fig. 5C) and siNC-transfected NBL-6 cells (Fig. 5D).

### Biliverdin mediates HO-1-induced anti-EqHV-8 effect

**Fig 6:**
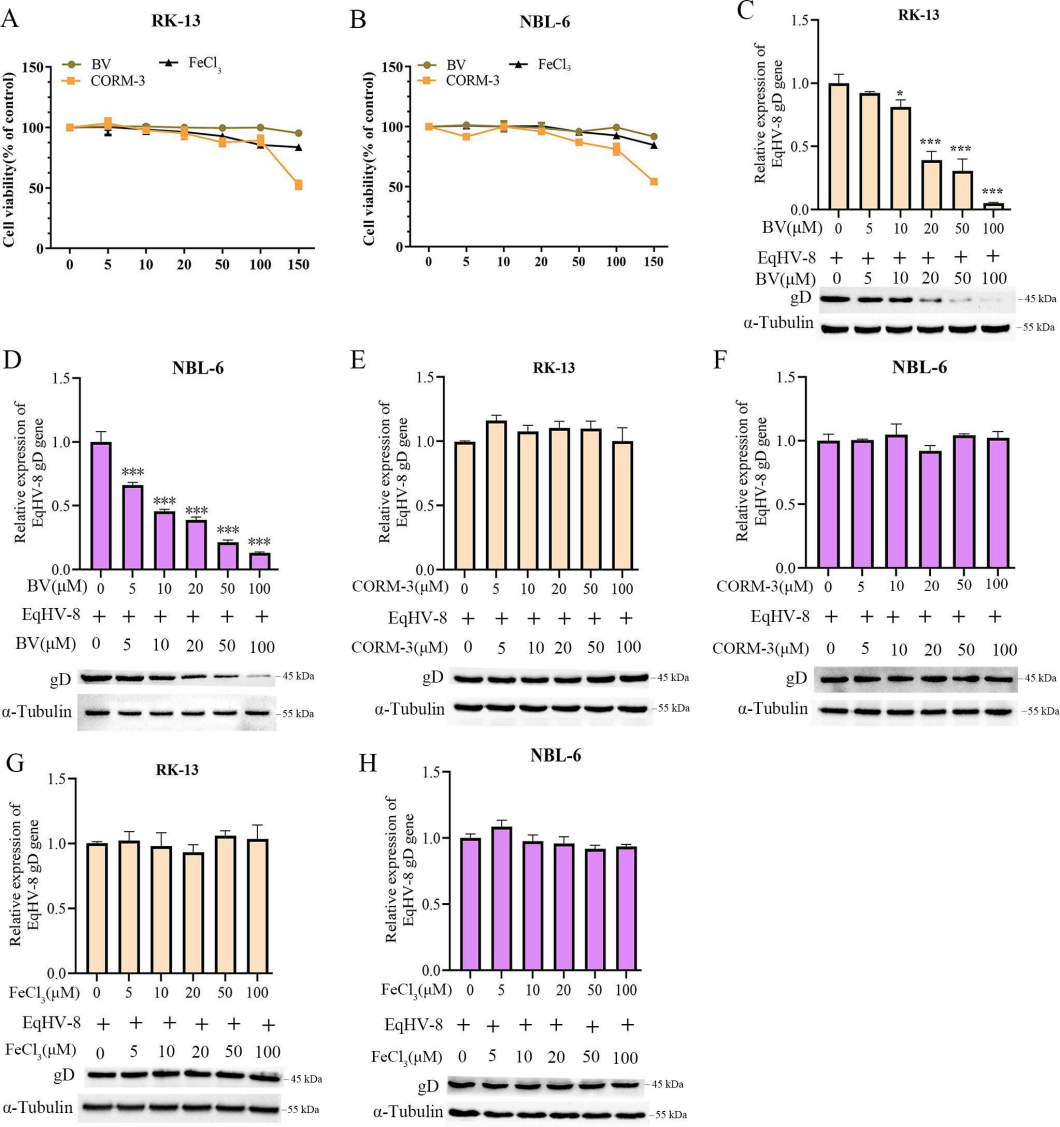
Biliverdin (BV) mediates the anti-EqHV-8 activity of HO-1. RK-13 (**A**) or NBL-6 (**B**) cells were treated with biliverdin, FeCl_3_, and CORM-3 at different concentrations for 24 h, and the cytotoxicity was detected by the CCK-8 assay. RK-13 (**C**) or NBL-6 (**D**) cells were pre-treated with biliverdin at different concentrations, followed by infection with EqHV-8 SDLC66 at an MOI of 0.1. These cells were collected to analyze the expression of gD at both mRNA and protein levels. Similar experiments were performed with CORM-3 (**E and F**) and FeCl_3_ (**G and **H) at indicated concentrations. The EqHV-8 replication was analyzed at 24 hpi by qPCR and Western blotting in RK-13 and NBL-6 cells. *GAPDH* served as the reference gene for qPCR, and α-tubulin acted as the loading control for Western blotting. The data are represented as mean ± SD from three independent experiments. *P < 0.05, ***P < 0.001 (compared with 0-µM BV, CORM-3, or FeCl_3_).

To investigate the mechanism of HO-1 against EqHV-8, we further assessed whether the HO-1 downstream metabolites, that is, BV, CO, and free iron, mediated the anti-EqHV-8 effect of HO-1. First, the cytotoxicity assay of BV, CORM-3, or FeCl_3_ was performed by CCK-8 in RK-13 and NBL-6 cells, and the data showed that BV and FeCl_3_ up to 150 µM and CORM-3 up to 100 µM did not affect the viability of RK-13 (Fig. 6A) and NBL-6 cells (Fig. 6B) cells. Next, RK-13 or NBL-6 cells were pre-treated with BV, CORM-3, or FeCl_3_ at different concentrations for 1 h, followed by infection with EqHV-8 SDLC66 for 24 h. qPCR and Western blotting were used to analyze the gD expression both at mRNA and protein levels. The results demonstrated that the gD expression of EqHV-8 decreased significantly in RK-13 (Fig. 6C) and NBL-6 (Fig. 6D) cells treated by BV in a concentration-dependent manner, compared with the untreated group. However, no changes were observed in gD expression in the RK-13-treated CORM-3 or FeCl_3_ group and control group (Fig. 6E and H) or NBL-6-treated CORM-3 or FeCl_3_ group (Fig. 6F and I). Our data indicated that BV, but not free iron and CO, mediated the HO-1-induced anti-EqHV-8 activity.

### BV mediates the anti-EqHV-8 effect via HO-1-induction and oxidative stress reduction

**Fig 7:**
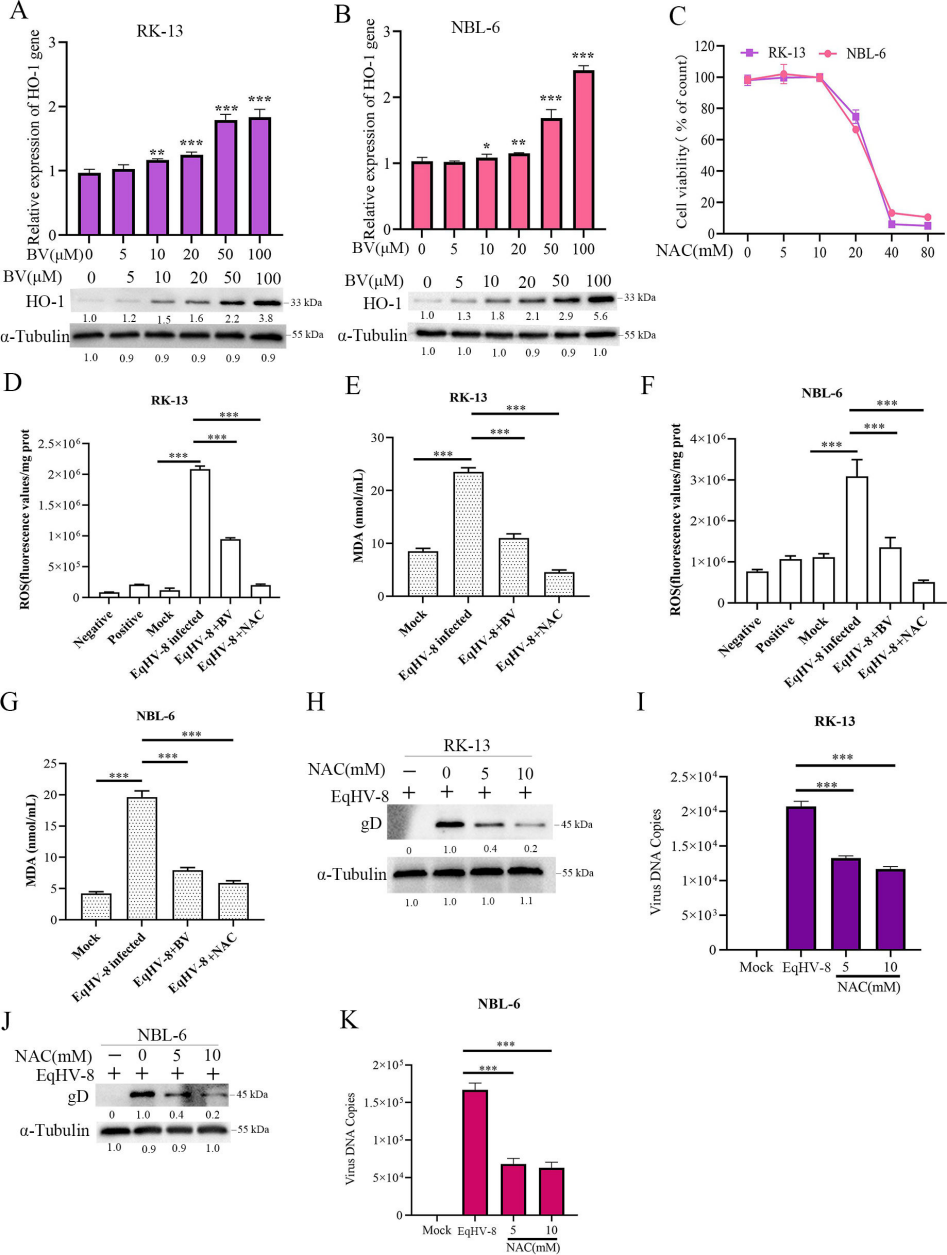
Biliverdin mediates the anti-EqHV-8 activity of HO-1 via reducing oxidative stress. RK-13 (**A**) or NBL-6 (**B**) cells were incubated by BV at different concentrations for 24 h and harvested to check HO-1 expression by qPCR and Western blotting. *P < 0.05, **P < 0.01, ***P < 0.001 (compared with 0-µM BV). The cytotoxicity of RK-13 or NBL-6 cells treated with different concentrations of NAC (0, 5, 10, 20, 40, and 80 mM) or Dimethyl sulfoxide DMSO (acted as 0-mM NAC) for 24 h was detected by the CCK-8 assay (**C**). RK-13 or NBL-6 cells were pre-treated with BV or NAC at indicated concentrations and infected with EqHV-8 at an MOI of 0.1. The cells were collected at 24 hpi to analyze ROS and MDA generation in RK-13 (**D and E**) and NBL-6 (**F and G**) cells using dichlorofluorescein or the MDA assay. The susceptible cells were treated with BV (100 µM) or NAC (10 mM), followed by infection with EqHV-8. The protein expression of gD and progeny virus copy number were detected at 24 hpi in RK-13 (**H and I**) or NBL-6 cells (**J and K**). Data are represented as mean ± SD from three independent experiments. ***P＜0.0001.

To assess whether the anti-EqHV-8 effect of BV was related to HO-1 expression in RK-13 and NBL-6 cells, these cells were treated with different concentrations of BV. The HO-1 expression was analyzed by qPCR and Western blotting. Our results showed a significantly elevated expression of HO-1 at both transcription and translation levels following BV treatment in a concentration-dependent manner as compared with the mock-treated RK-13 (Fig. 7A) or NBL-6 (Fig. 7B) cells. Biliverdin, an endogenous compound, has been reported to exert antioxidant and anti-inflammatory effects (34). Oxidative stress damage is common in virus-infected cells (18, 35). To explore if BV suppressed EqHV-8 replication by affecting oxidative stress reaction, we next evaluated the generation of ROS and malondialdehyde (MDA) in EqHV-8-infected RK-13 or NBL-6 cells. N-acetyl-L-cysteine (NAC) was used as an antioxidative agent; the maximum safe concentration of NAC was 10 mM (Fig. 7C). Our data showed that EqHV-8 infection increased the expression of ROS and MDA in RK-13 (Fig. 7D and E) and NBL-6 cells (Fig. 7F and G), and BV treatment significantly decreased EqHV-8-mediated ROS and MDA generation, which was consistent with the NAC treatment group (a positive control). In addition, NAC reduced the gD protein expression and progeny virus generation in RK-13 cells (Fig. 7H and I) and NBL-6 cells (Fig. 7J and K). These data indicated that the anti-EqHV-8 effect of BV largely depends on its antioxidative properties.

### BR mediates HO-1-induced anti-EqHV-8 activity by activating the PKCβ/ERK1/ERK2 signaling pathway

**Fig 8:**
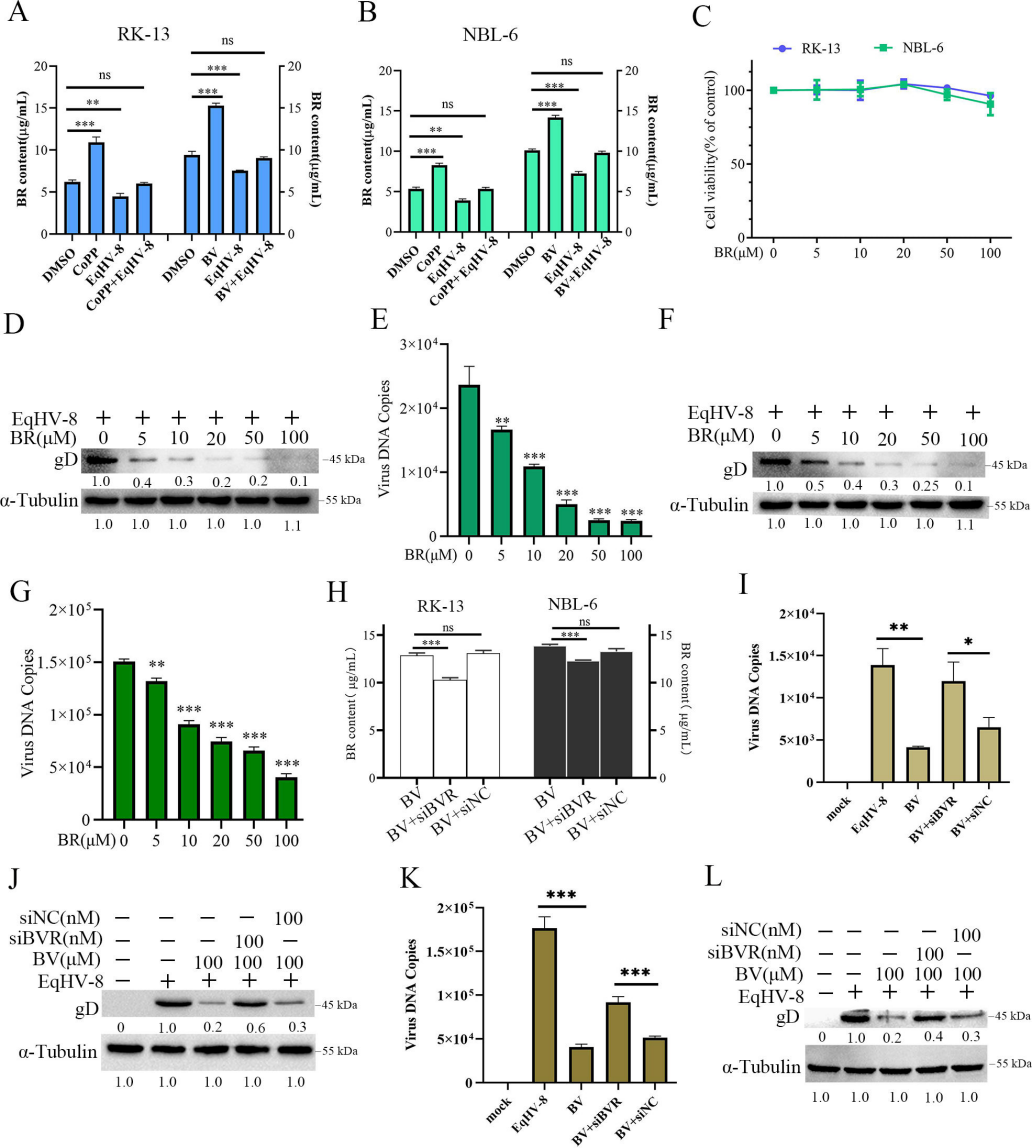
BV and its metabolite, BR, trigger a strong HO-1-mediated anti-EqHV-8 effect. RK-13 (**A**) or NBL-6 (**B**) cells were incubated in the presence or absence of CoPP (100 µM) or BV (100 µM), followed by infection with EqHV-8. Next, the cell supernatants were collected at 24 hpi to measure the BR contents with ELISA. RK-13 or NBL-6 cells were exposed to BR with 5, 10, 20, 50, and 100 µM or DMSO for 24 h, and the cell toxicity of BR was evaluated using the CCK-8 assay (**C**). The susceptible cells were treated with different concentrations of BR for 12 h and infected with EqHV-8 at an MOI of 0.1. The cells and cellular supernatants were harvested to analyze the protein expression of gD and progeny virus generation in RK-13 (**D and E**) and NBL-6 (**F and G**) cells. RK-13 or NBL-6 cells were transfected with siBVR or siNC at 100 nM for 12 h, followed by infection with EqHV-8 at an MOI of 0.1. The cell supernatants were harvested at 24 hpi to detect the generation of BR contents with ELISA (**H**). The siBVR or siNC was transfected into RK-13 and NBL-6 cells for 12 h, followed by infection with EqHV-8 for 24 h, and the expression of gD was analyzed by qPCR and Western blotting in RK-13 (**I and J**) and NBL-6 cells (**K and L**). *GAPDH* served as the reference gene for qPCR, and α-tubulin served as the loading control for Western blotting. Data are represented as mean ± SD from three independent experiments. *P < 0.05, **P < 0.01, ***P < 0.001. ELISA, enzyme-linked immunosorbent assay; ns, not significant; siBVR, siRNA targeting biliverdin reductase.

BR is a major downstream conversion product of BV and produces anti-viral effects in mammalian cells (17, 36). To test whether the change in the intracellular BR content in RK-13 or NBL-6 cells was related to EqHV-8 infection, these cells were incubated or unincubated with CoPP or BV at indicated concentrations, followed by infection with EqHV-8 for 24 h. As shown in Fig. 8A, CoPP or BV increased the BR content in RK-13 cells, and this activated effect was reversed by EqHV-8 infection. In addition, similar results were observed in NBL-6 cells (Fig. 8B). To further determine whether the anti-EqHV-8 effect of BV was mediated by BR, the cytotoxicity of BR in RK-13 cells or NBL-6 cells was first detected by CCK-8 assay. As revealed in Fig. 8C, BR up to 100 µM resulted in no change in these cells. The virus inhibition assay of BR at different concentrations was performed in RK-13 cells and NBL-6 cells. As observed in Fig. 8D, the protein level of gD decreased in the BR treatment group in RK-13 cells. The number of progeny viruses of BR-treated cells reduced significantly (Fig. 8E). A similar result was recorded in NBL-6 cells (Fig. 8F and G).

BVR has been implicated in modulating the transformation of BV into BR (37). To further evaluate whether specific siRNA targeting biliverdin reductase (siBVR) affected the anti-EqHV-8 effect of BV *in vitro*, RK-13 or NBL-6 cells were transfected with siBVR or siNC (100 nM) and infected with EqHV-8. The enzyme-linked immunosorbent assay (ELISA) results demonstrated that RK-13 transfected with siBVR partially decreased the conversion of BV to BR compared with the siNC-treated group (Fig. 8H). In addition, siBVR enhanced the protein and mRNA expression of gD (Fig. 8I and J) compared to the siNC-transfected group. Similar results were reported in NBL-6 cells (Fig. 8K and L).

**Fig 9:**
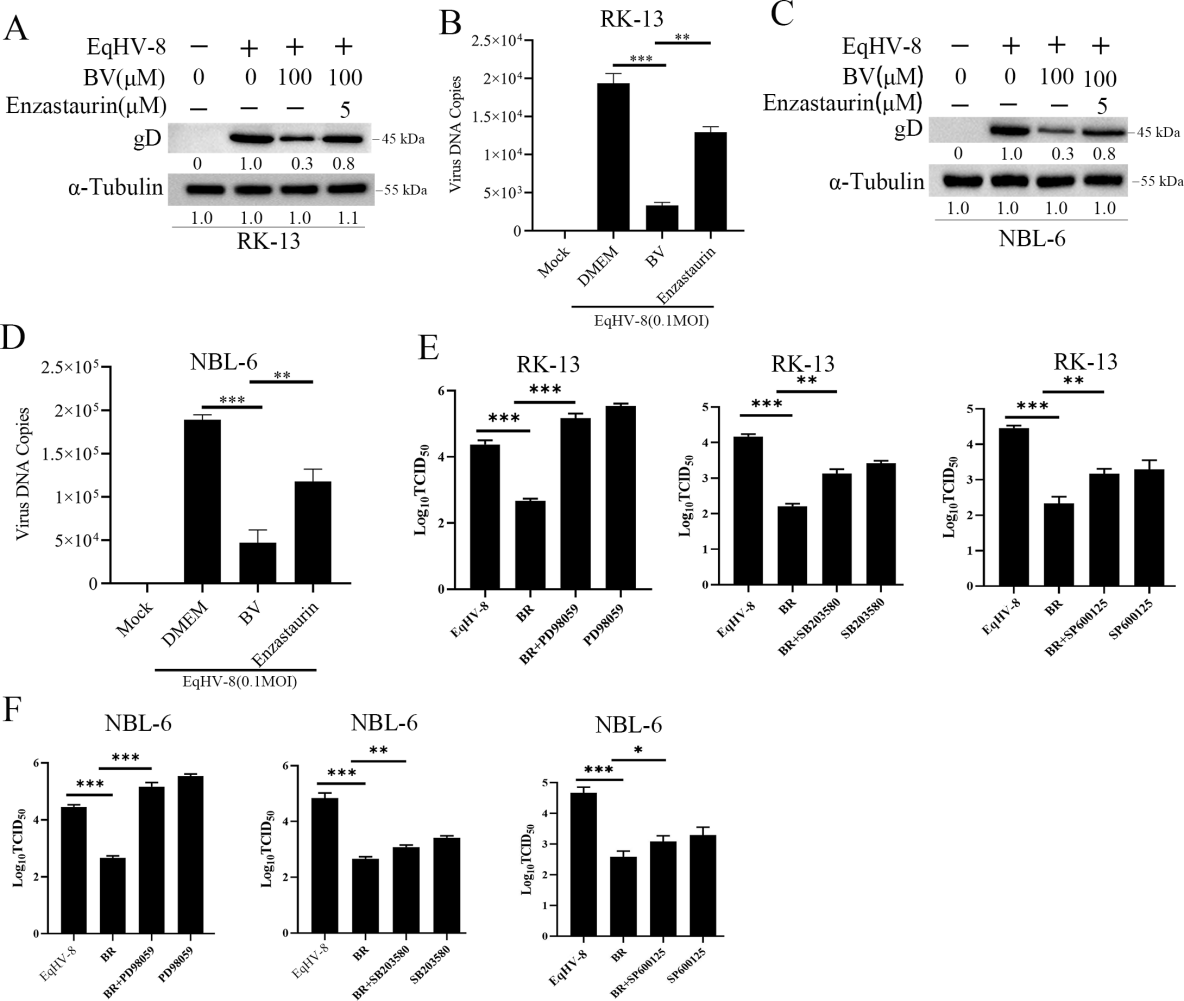
PKCβ and ERK1/ERK2 signaling pathways contribute to the anti-EqHV-8 effect of BV. The RK-13 or NBL-6 cells were pre-treated with Enzastaurin at 5 µM for 24 h, followed by EqHV-8 infection. The gD protein expression and progeny virus copy numbers were determined at 24 hpi in RK-13 (**A and B**) and NBL-6 (**C and D**) cells. The RK-13 (**E**) or NBL-6 (**F**) cells were treated with a mixture of BR (100 µM) and SB203580 (50 µM), PD98059 (50 µM), or SP600125 (50 µM), followed by EqHV-8 infection at 0.1 MOI. EqHV-8 replication was analyzed by TCID_50_. Data are represented as mean ± SD from three independent experiments. * *P* < 0.05, **P < 0.01, ***P < 0.001.

Previous studies reported that BVR, a multifunctional protein, plays an important role in cell survival through its antioxidant function via the PKCβ and MAPK signaling pathways (38, 39). Enzastaurin (PKCβ inhibitor) or different MAPK inhibitors, including p38 inhibitor SB203580, ERK1/ERK2 inhibitor PD98059, and JNK inhibitor SP600125, were used to assess if the anti-EqHV-8 effect of BV was related to the PKCβ or MAPK signaling pathways. We first determined the cell toxicity of PKCβ inhibitor or MAPK inhibitor in RK-13 cells and NBL-6 cells using the CCK-8 kit (Fig. S5). Our results demonstrated that the optimal concentration of enzastaurin reversed the anti-EqHV-8 effect of BV by gD expression and progeny virus copy number reduced in RK-13 cells (Fig. 9A and B). Similar results were observed in NBL-6 cells (Fig. 9C and D). Furthermore, RK-13 or NBL-6 cells were pre-treated with a mixture of BR (100 µM) and SB203580 (50 µM), PD98059 (50 µM), or SP600125 (50 µM) followed by infection with EqHV-8 at an MOI of 0.1. EqHV-8 replication was analyzed by TCID_50_ and Western blotting. As shown in Fig. 9E and Fig. S6A, PD98059 (ERK1/ERK2 inhibitor) reversed the anti-EqHV-8 effect of BV significantly in RK-13 cells. Similar results were observed in NBL-6 cells (Fig. 9F; Fig. S6B). However, no different changes were observed with other MAPK inhibitors such as SB203580 and SP600125 (Fig. 9E and F; Fig. S6A and B). These results indicated that the PKCβ/ERK1/ERK2 signaling pathway participates in BV-mediated and HO-1-induced anti-EqHV-8 effect.

### NO participates in the anti-EqHV-8 effect of BR in RK-13 and NBL-6 cells

**Fig 10:**
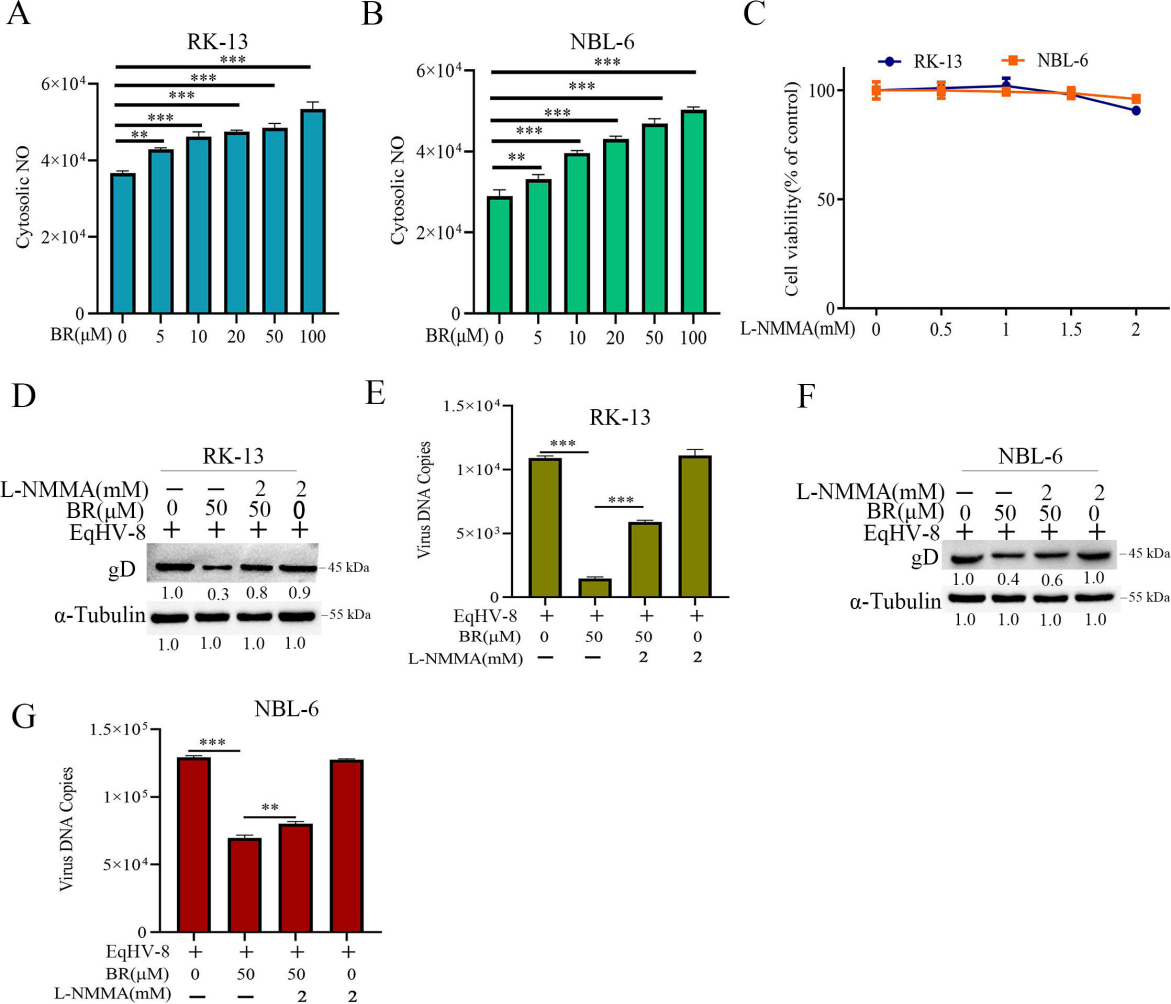
NO is involved in BR-mediated anti-EqHV-8 effect. RK-13 (**A**) or NBL-6 (**B**) cells were treated with different concentrations of BR (0, 5, 10, 20, 50, and 100 µM) for 24 h, followed by incubation with Dulbecco’s minimal essential medium without containing DAF-FMDA (5 µM/L) for 20 min. These cells were collected and washed with phosphate-buffered saline, and the NO generation was detected using the Tecan Spark microplate reader. The cell viability after treatment of RK-13 and NBL-6 cells with different concentrations of L-NMMA (0, 0.5, 1.0, 1.5, and 2.0 mM) was determined by CCK-8 assays (**C**). RK-13 or NBL-6 cells were incubated with a mixture of BR (50 µM) with or without L-NMMA (2 mM), followed by infection with EqHV-8 at an MOI of 0.1. The gD expression and virus copy number were determined in RK-13 (**D and E**) and NBL-6 cells (**F and G**). ** *P* < 0.01, ***P < 0.001.

Previous studies have demonstrated that BR can interact with NO and promotes its release by upregulating the expression of inducible nitric oxide synthase (40–42). To further explore whether the anti-EqHV-8 effect of BR depended on NO, we first detected the NO generation in RK-13 cells or NBL-6 cells by treating them with 0-, 5-, 10-, 20-, 50-, and 100-µM BR using 3-amino, 4-aminomethyl-2′,7′-difluorescein diacetate (DAF-FMDA). The results showed that BR significantly increased the intracellular NO generation in a concentration-dependent manner in RK-13 cells (Fig. 10A) and NBL-6 (Fig. 10B) cells. In addition, NG-monomethyl-L-arginine (L-NMMA), a NOS inhibitor, was used to confirm the effect of NO on the inhibition of EqHV-8 replication by BR. The cytotoxicity of L-NMMA on RK-13 cells or NBL-6 cells was determined by the CCK-8 assay, and our results showed that the maximum safe concentration of the L-NMMA was 2 mM (Fig. 10C). RK-13 cells or NBL-6 cells were treated by BR (50 µM) with or without co-incubation with L-NMMA (2 mM) and infected with EqHV-8 at an MOI of 0.1. The virus replication was analyzed at 24 hpi. The results demonstrated that the L-NMMA-treated group showed partially reversed BR-mediated anti-EqHV-8 activity, implying that the gD expression and progeny virus titers increased in RK-13 cells (Fig. 10D and E) and NBL-6 cells (Fig. 10F and G).

### Exogenous NO suppresses EqHV-8 replication *in vitro*

**Fig 11:**
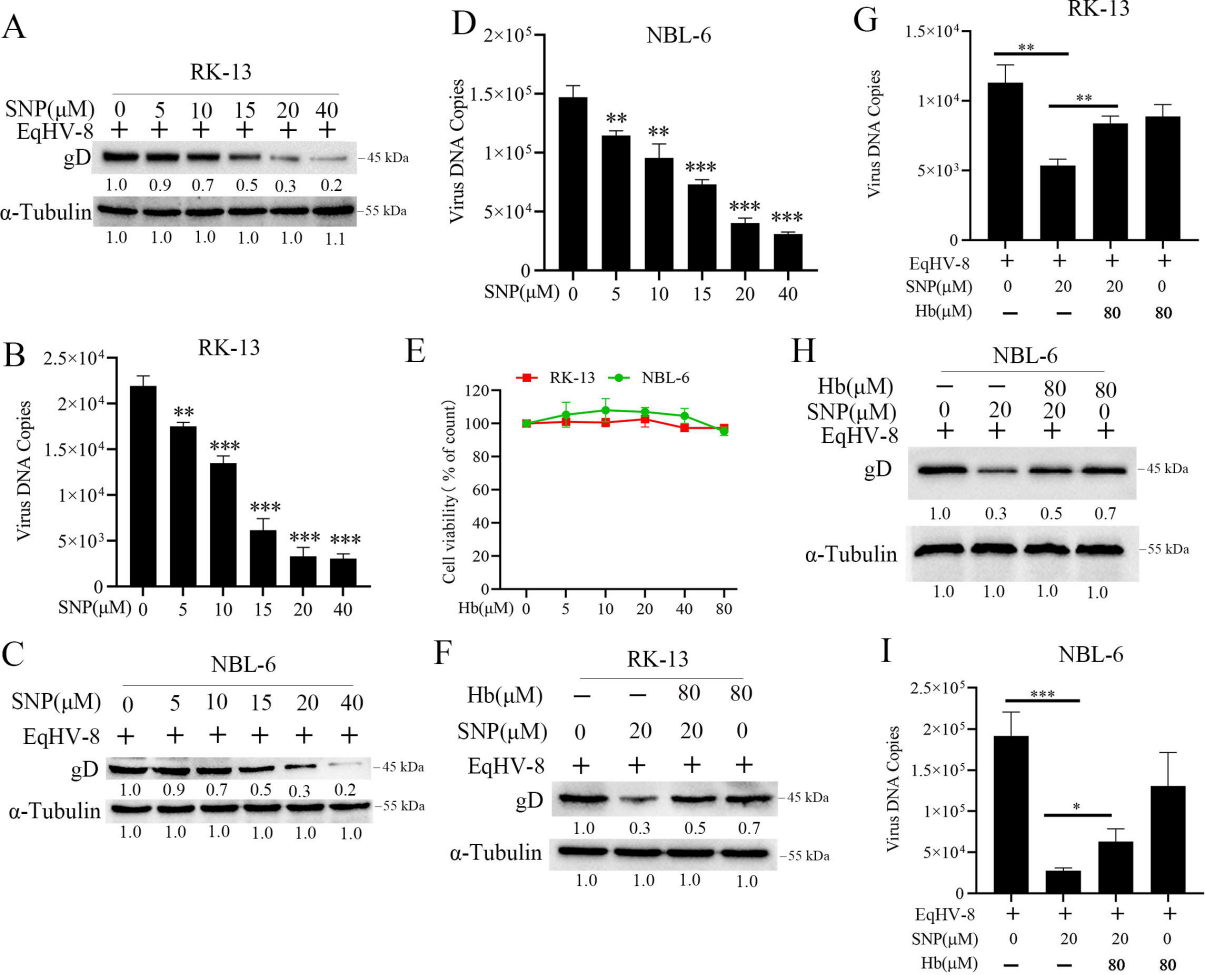
Exogenous NO suppresses EqHV-8 replication *in vitro*. RK-13 (**A and B**) or NBL-6 (**C and D**) cells infected with EqHV-8 at an MOI of 0.1 were treated with different concentrations of SNP (5, 10, 15, 20, and 40 µM). The cells and culture supernatants were harvested at 24 hpi. The gD protein expression and progeny virus generation were analyzed by Western blotting and qPCR. The cytotoxicity of RK-13 or NBL-6 cells treated with different concentrations of Hb (0, 5, 10, 20, 40, and 80 µM) for 24 h was detected by CCK-8 assay (**E**). Susceptible cells were pre-incubated in the presence and absence of Hb (80 µM) for 1 h, followed by infection of cells with EqHV-8 at an MOI of 0.1, and subsequent treatment with 20-µM SNP. EqHV-8 replication was detected in RK-13 (**F and G**) and NBL-6 (**H and I**) cells at 24 hpi by Western blotting and qPCR. Data are expressed as mean ± SD of three independent experiments. *P* values were calculated using Student’s *t*-test. *P < 0.05, **P < 0.01, and ***P < 0.001.

To further evaluate the anti-viral effect of NO against EqHV-8 infection in RK-13 or NBL-6 cells, single-nucleotide polymorphism (SNP), an exogenous NO donor, was used to treat RK-13 or NBL-6 cells at different concentrations (based on cytotoxicity assay as shown in Fig. S7) for 1 h after EqHV-8 infection. Next, the EqHV-8 gD protein expression and progeny virus generation were determined at 24 hpi by Western blotting and qPCR. As shown in Fig. 11A and B, SNP decreased the EqHV-8 replication in a concentration-dependent manner in RK-13 cells. Similar results were observed in NBL-6 cells (Fig. 11C and D). Subsequently, hemoglobin (Hb) (a specific NO scavenger) was applied to determine the anti-viral effect of SNP in RK-13 or NBL-6 cells. The cytotoxicity assay of Hb in RK-13 or NBL-6 cells was first determined using the CCK-8 kit. As shown in Fig. 11E, the maximum safe concentration of Hb was found to be 80 µM. Furthermore, the virus replication was detected by Western blotting and qPCR, which showed that Hb increased the gD protein expression and progeny virus production in RK-13 cells (Fig. 11F and G) and NBL-6 cells (Fig. 11H and I) compared to the Hb-untreated group. The finding that Hb reversed the anti-EqHV-8 effect of SNP by NO induction implied that NO plays a major role in BR-mediated anti-viral activity against EqHV-8 proliferation.

### NO-dependent cGMP/PKG participates in the anti-EqHV-8 effect of BR

**Fig 12:**
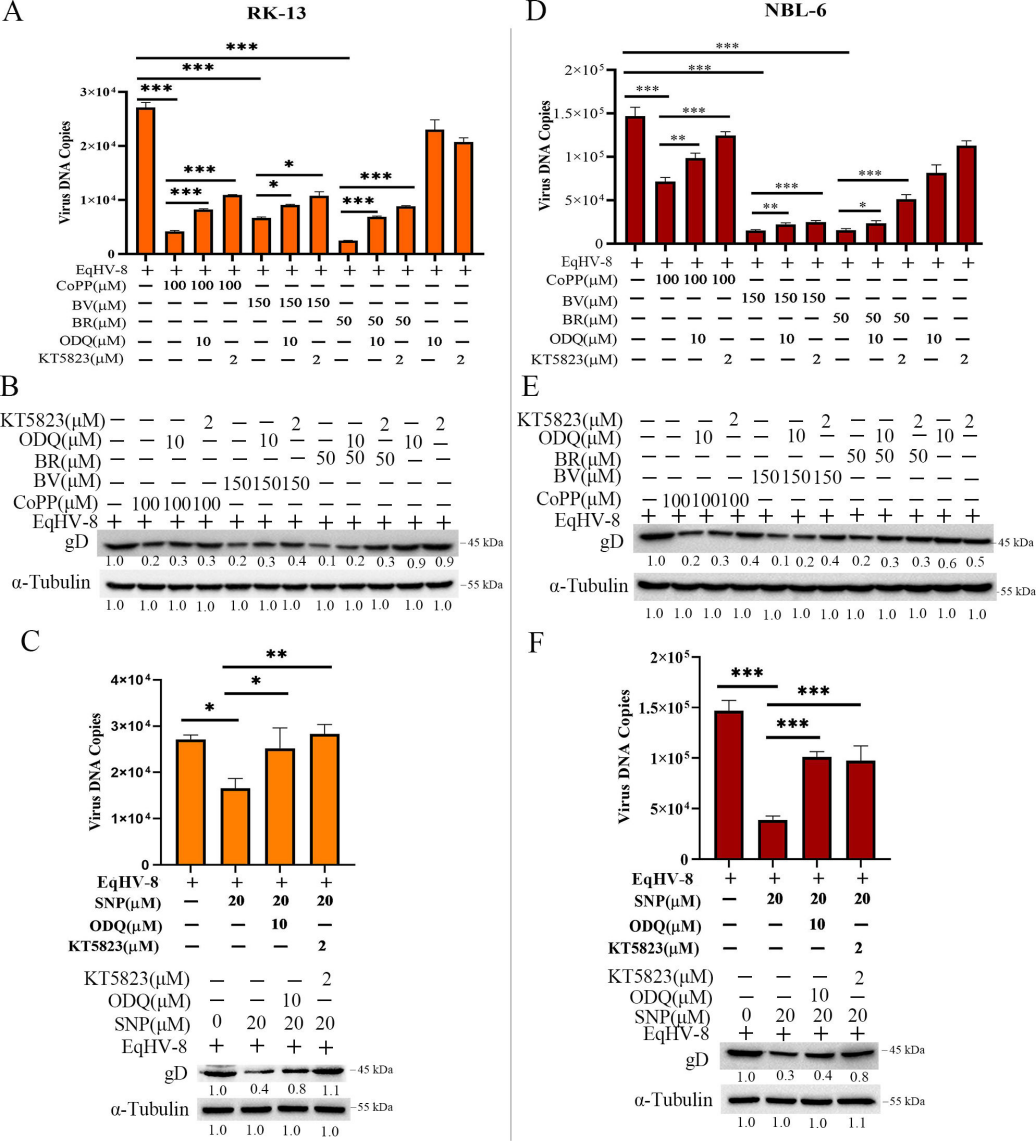
HO-1, BV, BR, and NO inhibition of EqHV-8 replication is mediated by the cGMP/PKG signaling pathway. The RK-13 or NBL-6 cells were infected with EqHV-8 at an MOI of 0.1 for 1 h, followed by the treatment of cells with or without CoPP (100 µM), BV (150 µM), BR (50 µM), and SNP (20 µM) in the presence or absence of an sGC-specific inhibitor ODQ (10 µM) or a PKG-specific inhibitor KT5823 (2 µM). The progeny virus copy number of EqHV-8 and gD protein expression was detected at 24 hpi in RK-13 (A–C) and NBL-6 cells (D–F) by qPCR and Western blotting. Data are expressed as mean ± SD of three independent experiments. *P* values were calculated using Student’s *t*-test. *P < 0.01, **P < 0.01, and ***P < 0.001.

Previous studies have reported that NO directly activates Soluble guanylyl cyclase (sGC) and increases the intracellular levels of cGMP in mammalian cells (43). Next, ODQ and KT5823 were used to check whether the NO-mediated anti-EqHV-8 mechanism was related to sGC activation or cGMP increase in the cGMP/PKG signaling pathway. The cellular cytotoxicity of ODQ or KT5823 on RK-13 or NBL-6 cells was evaluated using the CCK-8 kit (Fig. S8). The EqHV-8-infected RK-13 or NBL-6 cells were treated with CoPP (100 µM), BV (150 µM), BR (50 µM), and SNP (20 µM) in the presence or absence of ODQ (10 µM) or KT5823 (2 µM). The EqHV-8 infection was analyzed by qPCR and Western blotting. The results demonstrated that ODQ and KT582322 partially reversed the anti-viral effect of CoPP, BV, BR, and SNP by EqHV-8 progeny virus generation, and gD expression increased in RK-13 (Fig. 12A through C) and NBL-6 (Fig. 12D through F) cells. These data indicated the crucial role of the cGMP/PKG signaling pathway in NO-mediated anti-viral effect against EqHV-8 infection.

### HO-1 decreases EqHV-8 replication in the lungs of mice model

**Fig 13:**
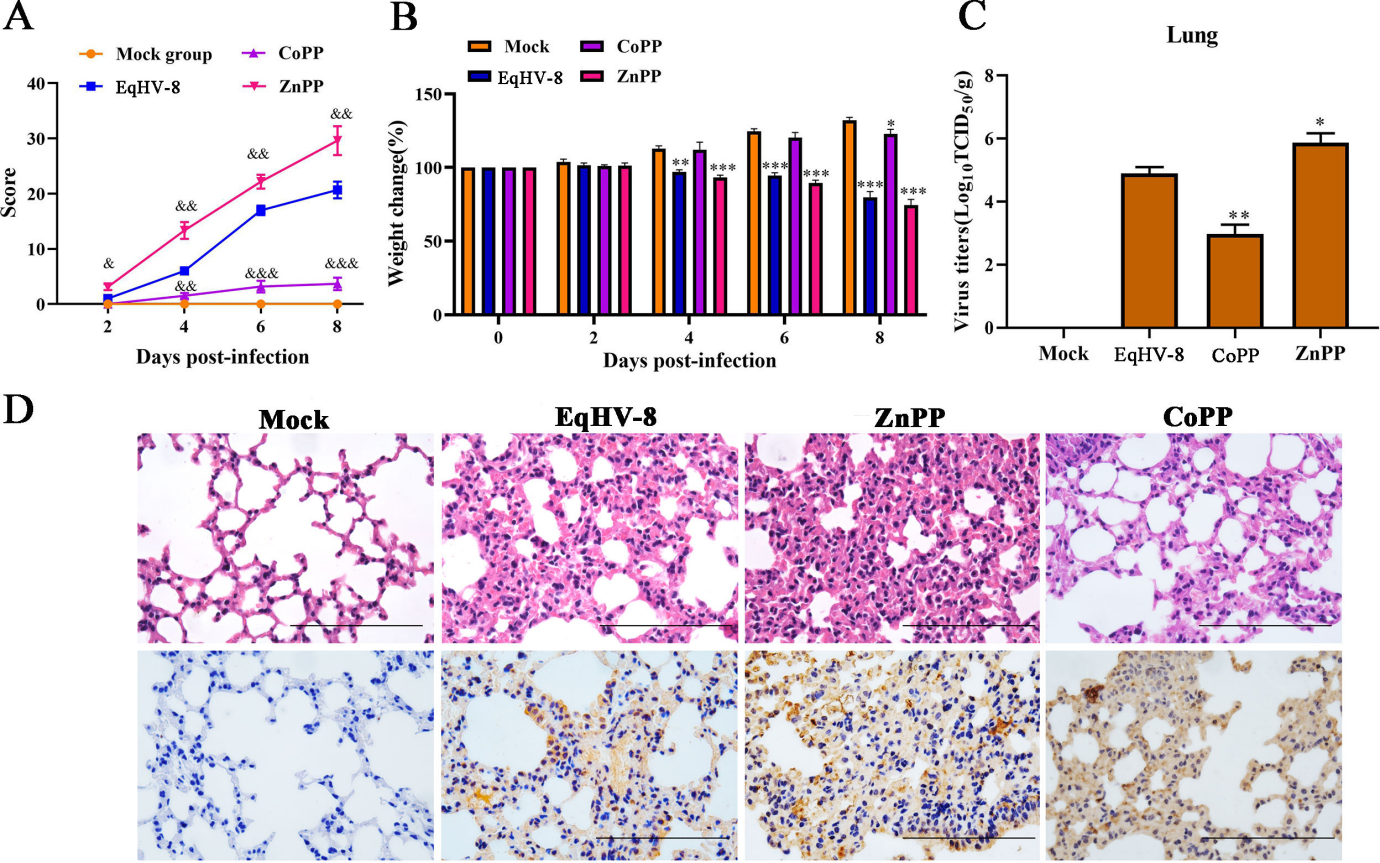
HO-1 serves as an anti-viral factor and suppresses EqHV-8 replication in mice. Twenty mice were randomly divided into four groups, namely, mock group, EqHV-8-infected group, EqHV-8 with CoPP group, and EqHV-8 with ZnPP group. Clinical symptoms (**A**) and body weight (**B**) of mice were monitored and scored at indicated time points. ^&^*P* < 0.05, ^&&^*P* < 0.01, ^&&&^*P* < 0.001 compared with the EqHV-8 group, *P < 0.05, **P < 0.01; ***P < 0.001 compared with the mock group. (**C**) The lung tissues of different groups were collected at 8 dpi to detect EqHV-8 replication by titrating in the RK-13 cells. *P < 0.05, **P < 0.01, compared with the EqHV-8-infected group. (**D**) Representative images of hematoxylin and eosin staining and immunohistochemistry (for EqHV-8 using the positive serum in the lungs derived from the mock, EqHV-8, CoPP, or ZnPP-groups. Scale bar, 100 µm.

The BALB/c mice have been used to evaluate virus replication and virulence of EqHV-8 as described previously (44). To further confirm the effect of HO-1 against EqHV-8 infection *in vivo*, 20 BALB/c mice were randomly divided into four groups. Mice were injected intraperitoneally with ZnPP or CoPP, followed by intranasal inoculation with EqHV-8. Mice demonstrated depression, dyspnea, and hair shedding starting from 2 dpi, with clinical symptom scores as shown in Fig. 13A. The body weight of mice had markedly decreased from 4 dpi in ZnPP and EqHV-8 groups compared with the mock group, and ZnPP caused more severe weight loss. However, intraperitoneal injection of CoPP alleviated the clinical symptoms caused by virus infection (CoPP-treated group), where mice showed increased body weight, with no observed difference compared with the mock group, excluding those at 8 dpi (Fig. 13B). The lung tissues were collected and ground to evaluate EqHV-8 replication in RK-13 cells. The results showed that the mean titers of EqHV-8 in the CoPP-treated group were suppressed significantly compared to those in the EqHV-8 group. In contrast, ZnPP treatment significantly promoted EqHV-8 replication (Fig. 13C). For histopathology, the lungs of BALB/c mice treated with CoPP showed moderate-to-mild alveolar wall thickening and mild infiltration of inflammatory cells compared with mice in the EqHV-8 group. However, ZnPP is more able to aggravate the tissue damage caused by EqHV-8 infection, with no significant histopathological changes observed in the lungs of mice in the mock group (Fig. 13D). Consistent with this, Consistently, less viral antigen-positive cells were present in the lungs of mice in the CoPP-treated EqHV-8 group than that in the EqHV-8-infected mice at 8 dpi; however, ZnPP increased viral antigen-positive cells compared with those in EqHV-8-infected mice lungs (Fig. 13D). These data suggested that HO-1 served as a host anti-viral factor against EqHV-8 infection *in vivo*.

## DISCUSSION

**Fig 14:**
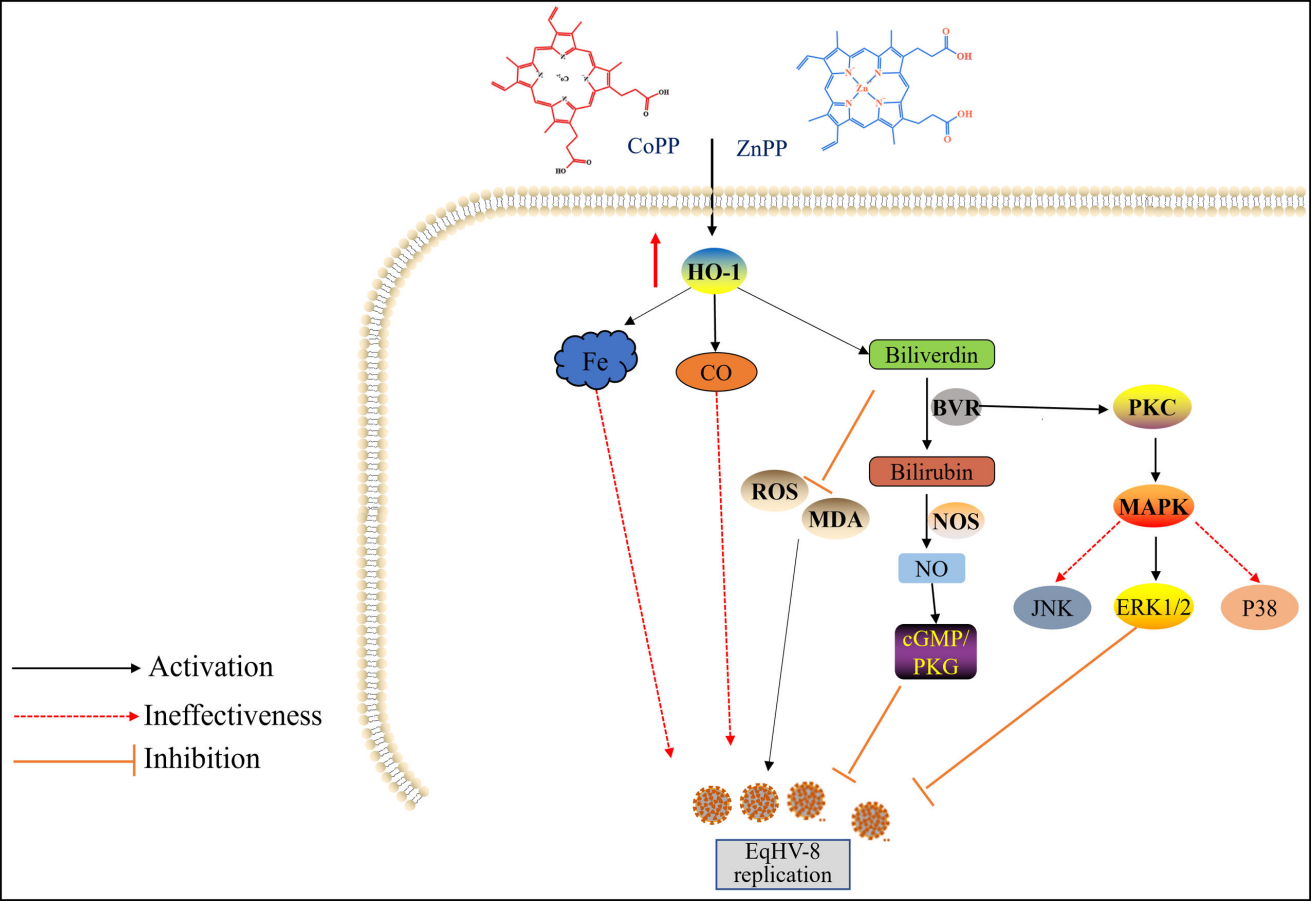
Scheme depicting the mechanism of HO-1 and its metabolites against EqHV-8 infection. EqHV-8 infection decreases endogenous HO-1 expression, and EqHV-8 infection efficiency is negatively correlated with HO-1 expression. Three metabolites, namely, BV, CO, and iron, are produced by HO-1 degradation. BV, not CO or iron, suppresses EqHV-8 replication by reducing ROS production by activating the PKCβ and ERK1/ERK2 signaling pathways. Subsequently, BV is converted into BR by the BVR enzyme. BR induces NO generation to reduce EqHV-8 infection through the activation of the NO-dependent cGMP/PKG signaling pathway.

EqHV-8 is a virus of the Herpesviridae family with a double-stranded enveloped DNA. It belongs to the subfamily Alphaherpesvirinae (45). It was first isolated from the nasal cavity of latently infected donkeys in Australia in 1988 (46) and subsequently found in a horse with fever and runny nose from China, and in a donkey from Israel (47, 48). Recently, our group has confirmed that EqHV-8 is closely related to abortion, respiratory diseases, and viral encephalitis in donkeys (2, 3, 44, 49). It is highly crucial to develop novel anti-viral strategies to control the EqHV-8 outbreak. In the present study, we determined the role of HO-1 in EqHV-8 replication and explored the molecular mechanism underlying the anti-EqHV-8 effect of HO-1. Our results demonstrated that EqHV-8 infection decreased the HO-1 expression in both RK-13 and NBL-6 cells, and the EqHV-8 replication was reduced or increased with HO-1 induction or knockdown, respectively. Furthermore, BV, an HO-1 downstream metabolite, mediates its anti-viral effects by reducing the levels of ROS and MDA. These processes are regulated by the PKCβ/ERK1/ERK2 and NO-dependent cGMP/PKG pathways (Fig. 14). In addition, we confirmed that HO-1 exerts its anti-viral effect against EqHV-8 infection using a mouse model (Fig. 13).

HO-1 has been implicated in several virus infections. In addition, the overexpression of HO-1 is known to suppress multiple viral infections (11, 15–17, 50). These metabolites of HO-1 have been reported to have anti-inflammatory and antioxidant effects and to protect tissues or organs against several pathogens. However, the change in the expression of HO-1 depends on different virus infections, for example, hepatitis B virus, HCV, Ebola virus, DENV, PRV, and PCV3, and reduces HO-1 expression in host cells during infection (18). To investigate the role of HO-1 during EqHV-8 infection in the present study, we demonstrated that the HO-1 expression was downregulated during EqHV-8 replication in RK-13 and NBL-6 cells at 12–48 hpi (Fig. 1), which was consistent with the findings of previous studies (17). Furthermore, CoPP- or piggyBac transposon system-mediated HO-1 expression was significantly reduced following EqHV-8 infection in susceptible cells (Fig. 2 and 4). Meanwhile, HO-1 activity decreased with ZnPP or siRNA in RK-13, and NBL-6 cells increased EqHV-8 infection (Fig. 3 and 5). In addition, BALB/c mice were injected intraperitoneally with CoPP or ZnPP to assess the HO-1-mediated anti-viral effect against EqHV-8 replication *in vivo*. Our results demonstrated that CoPP improved clinical symptoms and reduced EqHV-8 replication in lung tissues (Fig. 13), which was consistent with *in vitro* results. These data suggested that the enzyme activity of HO-1 was essential for EqHV-8 inhibition, and the expression of HO-1 negatively regulates EqHV-8 replication in host cells.

It has been previously reported that the anti-viral role of HO-1 is intricately associated with the metabolites of HO-1, including BV, CO, and iron. These metabolites of HO-1 were reported to have anti-inflammatory and antioxidant effects and to protect tissues or organs against several pathogens (9, 51–53). For example, Zhang et al. demonstrated that HO-1 metabolites BV and CO, but not iron, inhibit PRRSV or PRV replication *in vitro* (17, 27). Wang et al. reported that the ferric ions, but not BV and CO, mediated the HO-1 effect against duck Tembusu virus infection (51). In the present study, the anti-EqHV-8 molecular mechanisms mediated by HO-1 were explored *in vitro*, and our results revealed that when compared with the control group, only BV could effectively inhibit the replication of EqHV-8, whereas CORM-2 and FeCl_3_ did not exert an anti-EqHV-8 effect *in vitro* (Fig. 6).

Oxidative stress injury often occurs in a virus-infected cell, HO-1, and its metabolite BV was found to possess an antioxidative activity by a ROS scavenger (18, 27). Our results demonstrated that EqHV-8 infection induced ROS and MDA generation in RK-13 and NBL-6 cells, whereas BV reduced ROS and MDA expression by upregulating the expression of HO-1 (Fig. 7A through E). Moreover, NAC (a ROS inhibitor) significantly inhibited EqHV-8 infection by decreasing the intracellular ROS levels in RK-13 and NBL-6 cells (Fig. 7E through H). These data suggested the BV-mediated anti-EqHV-8 activity was dependent on ROS generation.

Bilirubin, which is both cytoprotective and cytotoxic, quenches ROS and inhibits inflammatory and mitogen-induced ROS-mediated responses (54). Previous studies have reported that BV is involved only in the metabolic pathway to BR via the production of BR via BVR (55). Thus, BVR plays a crucial role in cellular defense mechanisms. We further checked the anti-EqHV-8 effect of BR. We demonstrated CoPP or BV treatment induced the generation of BR in RK-13 and NBL-6 cells (Fig. 8A and B), and BR exerted an anti-EqHV-8 effect at different concentrations (Fig. 8D through G). The anti-EqHV-8 activity of BV was reversed by siBVR treatment, which reduced the production of BR (Fig. 8H through L), suggesting the crucial function of BR BV against EqHV-8 infection. This phenomenon was also consistent with a previous study in PRRSV or PCV3 (18, 27).

BVR has been implicated in antioxidant functions, which are essential for PKCβ and MAPK signaling pathways (37). Next, we evaluated whether the anti-EqHV-8 effect of BV was associated with PKCβ or MAPK signaling pathways with specific inhibitors. Our results demonstrated that enzastaurin (PKCβ inhibitor) reversed the anti-EqHV-8 effect of BV *in vitro* (Fig. 9A through D). Moreover, PD98059 (ERK1/ERK2 inhibitor) reduced the anti-EqHV-8 effect of BV in RK-13 and NBL-6 cells (Fig. 9E and F; Fig. S6). These data suggested that BV exerted an anti-viral effect by activating the PKCβ and ERK1/ERK2 signal pathways.

BR is known to induce NO production in the brain through neuronal NO synthase (56). NO mediated the anti-viral effect of BR in PCV3 and PRRSV infection, as previously described. Furthermore, we explored the inhibitory mechanisms of BR targeting EqHV-8. Our data showed that BR increased the generation of NO *in vitro* (Fig. 10A and B). L-NMMA (a total NOS inhibitor) partially attenuated the anti-EqHV-8 activity of BR-induced NO in RK-13 and NBL-6 cells (Fig. 10D through G). Our data suggested that BR suppressed EqHV-8 replication by increasing the expression of NO. In addition, the NO inducer (SNP) or scavenger (Hb) was used to confirm the anti-EqHV-8 activity of exogenous NO (Fig. 11).

Previous studies have implicated NO in activating guanylate cyclase, a heme-containing enzyme. In addition, NO interacted with the cytosolic form of guanylate cyclase to induce the production of cGMP. Our data demonstrated that ODQ or KT5823, cGMP/PKG pathway inhibitors, reduced the anti-EqHV-8 effect mediated by CoPP, BV, BR, or SNP (Fig. 12). These results suggested that HO-1 metabolite BV/BR exhibited the anti-EqHV-8 effect by activating the PKCβ/ERK1/ERK2 signaling pathway. Moreover, the HO-1 metabolite NO exerted anti-EqHV-8 activity in a cGMP/PKG-dependent signaling pathway manner (Fig. 14).

In summary, our study demonstrated that HO-1 is a potent host defense factor which exerts anti-viral activity against EqHV-8 infection. These functions are at least partially mediated by its downstream metabolite, BV, via the activation of the PKCβ/ERK1/ERK2 and NO-dependent cGMP/PKG signaling pathways. These results suggested that HO-1 could be developed as a potential novel anti-viral agent for controlling EqHV-8 infections.

## MATERIALS AND METHODS

### Cell lines, viruses, chemicals, and antibodies

RK-13 cells were purchased from the China Center for Type Culture Collection (Wuhan, China) and maintained in Dulbecco’s minimal essential medium (DMEM; Life Technologies Corporation, Carlsbad, USA) containing 10% fetal bovine serum (FBS; Gibco, Grand Island, USA) at 37°C and 5% CO_2_. Equine dermal (NBL-6) cells were purchased from the American Type Culture Collection (Manassas, USA) and cultured in 10% FBS Eagle’s minimum essential medium (MEM, Life Technologies Corporation). RK-13 cells were used to proliferate and titrate the EqHV-8 SDLC66 (GenBank: MW816102.1).

CoPP (HO-1 inducer), BV, BR, FeCl3, CORM-3, and PD 98059 (ERK1/ERK2 inhibitor), SP600125 (JNK inhibitor), (OC-6–44)-tricarbonylchloro (glycinato) ruthenium (CORM-3), and SB203580 (p38 inhibitor) were purchased from Sigma (St. Louis, MO, USA). Enzastaurin and CCK-8 were obtained from Sparkiade Biotechnology Co. Ltd (Jinan, China). KT5823 (PKG inhibitor), ZnPP (HO-1 enzyme activity inhibitor), and ODQ (sGC inhibitor) were purchased from Med Chem Express (MED, New Jersey, USA). DAF-FMDA, 2′,7′-dichlorodihydrofluorescein diacetate (DCFH-DA), Hb, sodium nitroferricyanide (III) dihydrate (SNP, an NO donor), NAC (a ROS inhibitor), and L-NMMA, a NOS inhibitor) were obtained from Beyotime (Nanjing, China). BR ELISA kit was purchased from Elabscience (Wuhan, China). Mouse anti-EqHV-8-positive serum and mouse anti-gD (envelope glycoprotein D) polyclonal antibody were prepared in our laboratory. Mouse anti-HO-1 antibody and anti-tubulin antibody were purchased from Abcam (Cambridge, MA, USA).

### Cell viability assay

The CCK-8 assay was performed to evaluate the cytotoxicity of different inhibitors in RK-13 and NBL-6 cells as described previously (57). Briefly, cells were seeded into a 96-well plate (1 × 10^4^/well), to which different concentrations of each of the chemical drugs were added after 24 h and then maintained for 24 h. The CCK-8 reagent (10 µL/well) was added for another 2 h. The emitted light at 450 nm was measured by the Epoch Microplate spectrophotometer (BioTek, Winooski, Vermont, USA), and the 50% cytotoxic concentration (CC50) value was analyzed with GraphPad Prism version 8.

### IFA

RK-13 and NBL-6 cells were seeded on coverslips in 12-well cell culture plates overnight, pre-incubated with 0-, 5-, 10-, 15-, and 20-µM ZnPP for 12 h, and infected with EqHV-8 SDLC66 (MOI of 0.1 ) for 1 h. The medium was changed with 3% FBS DMEM with ZnPP at indicated concentrations. The cells were fixed with 75% cold ethanol at 36 hpi, following which they were blocked with 1% bovine serum albumin (Solarbio, Beijing, China) in phosphate-buffered saline (PBS). The cells were next incubated with mouse anti-EqHV-8-positive serum and later with rhodamine-conjugated goat anti-mouse IgG secondary antibody. Finally, the cells were stained with 4′,6-diamidino-2-phenylindole (Sigma) and visualized using Leica microsystems (Leica DMi 8, Wetzlar, Germany).

### qPCR

All cell samples were collected at 24 hpi for qPCR analysis, which was performed on a Step One Plus real-time PCR system as previously described (58). Briefly, the total RNA was extracted from cells using TRIzol reagent (Invitrogen, Carlsbad, CA, USA) in accordance with the manufacturer’s instructions. The RNA was reverse transcribed to cDNA using PrimeScript RT Master Mix kit (Takara, Tokyo, Japan). The primers of HO-1 and gD gene are listed in Table 1, and the transcripts of glyceraldehyde dehydrogenase or β-action were amplified to normalize the total RNA input. The relative quantification of target genes was performed using the 2−ΔΔCt method.

**TABLE 1:** The primers in this study^*a*^

Primers	Primer sequences (5′−3′）
HO-1-F	CTAGCTAGCATGGAGCACCCGCAGCAGCC
HO-1-R	CGCGGATCCCATGGCATAGAGCCCCACGGC
^q^HO-1-F	AGTTCATGAAGAACTTTCA
^q^HO-1-R	TACCAGAAGGCCATGTCC
ORF72-F	CCCACGTGTGCAACGCCTAT
ORF72-R	ATACAGTCCCGAGGCAGAGT
GAPDH-F	CCTTCCGTGTCCCTACTGCCAAC
GAPDH-R	GACGCCTGCTTCACCACCTTCT
^H^β-action	ACGGCATCGTCACCAACTG
^H^β-action	CAAACATGATCTGGGTCATCTTCTC

^*a*^ ^q^HO-1 was used for qPCR. ^H^β-action was used for NBL-6 cells.

To determine the EqHV-8 genome DNA copy numbers, absolute quantification PCR was performed with pMD18-T-gD as the template, and a fragment of gD (ORF72 gene), 186 bp in size, was cloned into the pMD18-T vector to generate recombinant plasmid, that is, pMD18-T-gD. It served as the standard sample to calculate the EqHV-8 genome DNA copy number.

### Western blotting analysis

Cells were collected and lysed with NP-40 cell lysis buffer (Solarbio) and mixed with the 5× sample loading buffer for sodium dodecyl sulfate–polyacrylamide gel electrophoresis (SDS–PAGE). The samples were loaded onto 12% SDS–PAGE gels with equal amounts, and the separated proteins were further transferred onto polyvinylidene fluoride (PVDF; Merck Millipore, Billerica, MA, USA) membranes as described previously (59). The PVDF membranes were blocked with 5% non-fat dry milk and incubated with anti-HO-1 mAb, anti-α-tubulin mAb, or anti-EqHV-8 gD polyclonal antibody. The specific binding of antibodies to their targets was detected with horseradish peroxidase-conjugated secondary antibodies, either goat anti-mouse or goat anti-rabbit IgG (Invitrogen). Finally, the images of membranes were analyzed with the ChemiDoc XRS imaging system (Bio-Rad, Hercules, CA, USA).

### Virus titration

RK-13, RK-13^HO-1^, and RK-13^Vector^ were plated into six-well cell plates at a density of 2 × 10^5^ cells per well overnight. Next, these cells were infected with EqHV-8 SDLC66 at an MOI of 0.1 for 1 h, and the medium was replaced with 3% FBS MEM for 24 h. The cell and cellular supernatant were frozen and thawed thrice, and collected to measure the virus progeny titer as follows. The same operation was performed for NBL-6^HO-1^ and their parent cells.

The production of virus progeny was studied using RK-13 cells following the Reed–Muench method as described previously (2). Briefly, the RK-13 cells were seeded into 96-well plates 24 h before virus infection. Next, the viral supernatant was serially diluted 10-fold in eight replicates with 100-µL/well addition. After 5 days post-infection, the TCID_50_ was calculated and analyzed with GraphPad Prism version 8.0.

### Modulation of HO-1 activity and EqHV-8 replication

To check whether the HO-1 enzyme activity was necessary for its anti-viral function, RK-13 or NBL-6 cells were pre-treated with ZnPP or CoPP with different concentrations at 37°C for 12 h, respectively, followed by infection with EqHV-8 at an MOI of 0.1. These cells were collected to study gD protein expression via Western blotting and IFA. The production of progeny viruses from cellular supernatant was detected by qPCR.

### Plasmid construction and generation of stable cell lines

The total RNA was extracted from donkey lung tissues using TRIzol assay and reverse transcribed into cDNA with PrimeScript RT Master Mix as described above. The donkey HO-1 gene was amplified with specific primers (listed in Table 1) and cloned into the piggyBac Transposon vector system (System Biosciences, USA) using *Nhe* I and *Bam* HI restriction enzymes. RK-13 and NBL-6 cell lines stably expressing HO-1 were generated as described previously (60). Briefly, the RK-13 cells were seeded into 12-well plates at a density of 1 × 10^5^ cells/well. When cells reached 70%–80% confluency, they were co-transfected with donor plasmid pB-HO-1 and helper plasmid PA using Lipofectamine 6000 transfection reagent (Beyotime). These cells were cultured with a puromycin-selective medium (10 µg/mL), and positive cell colonies with green fluorescent protein were observed by an inverted microscope and checked by Western blotting.

### siRNA knockdown experiments

The siRNAs targeting HO-1 or BVR gene were designed and chemically synthesized by RiboBio Co., Ltd. (Guangzhou, China), which was listed in Table 2. The siRNA knockdown assay was performed as previously described (61). Briefly, RK-13 and NBL-6 cells were transfected with specific siRNAs using Lipofectamine 6000 according to the manufacturer’s protocol for 12 h, followed by infection with EqHV-8 at an MOI of 0.1 and incubation with CoPP or BV. Cell samples and cell supernatants were collected at the indicated times to analyze HO-1 expression, virus replication, and BR generation using qPCR, Western blotting, TCID_50_, or ELISA.

**TABLE 2:** The sequence of siRNA used in this study

Primers	Sequences (5′−3′）
siHO-1-①	GGTCCTCACACTCAGCTTT
siHO-1-②	CCACCAAGTTCAAGCAGCT
siHO-1-③	CCACCAAGTTCAAGCAGCT
siBRV-①	CTACATCAGGCAGTTTCTT
siBRV-②	CCAAATGTAGGCGTCAATA
siBRV-③	GGCGTTCCTGAACCTGATT

### ELISA detection of intracellular BR

The RK-13 or NBL-6 cells were cultured into six-well cell plates and incubated with the indicated concentrations of CoPP or BV, followed by infection with EqHV-8 (0.1 MOI). Cells were collected at 24 hpi and lysed in NP40 lysis buffer. The supernatants were obtained to detect the intracellular BR with a BR ELISA kit according to the manufacturer’s protocol. The absorbance was measured at 450 nm using the Epoch Microplate spectrophotometer (BioTek).

### ROS, MDA, and NO detection

The cellular levels of ROS generation in RK-13 or NBL-6 cells with EqHV-8 infection were measured using the ROS detection kit (Jiancheng, Nanjing, China) according to the manufacturer’s instructions. Briefly, RK-13 or NBL-6 cells were seeded into 12-well plates, pre-incubated with 100 µM BV for 2 h, followed by infection with 0.1 MOI EqHV-8 for another 1 h. Next, the medium was replaced with 3% FBS MEM for 24 h. After the addition of DCFH-DA (10 µM/L), the plates were incubated at 37°C for 20 min and washed thrice with PBS. These cells were collected to measure the fluorescent intensity using the Spark microplate reader (Tecan, Switzerland). The cells were treated with NAC (N-acetyl-L-cysteine) as the positive control. Finally, the data were analyzed by GraphPad Prism.

The levels of MDA in EqHV-8-infected RK-13 or NBL-6 cells were determined using the Microscale MDA) assay kit according to the manufacturer’s instructions (Jiancheng). The levels were normalized to the protein concentration determined by a Pierce BCA protein assay kit (Thermo Fisher Scientific, Waltham, MA, USA). These data were determined by ultraviolet–visible spectrophotometer (UV-8000, Shanghai, China).

The intracellular NO generation in RK-13 or NBL-6 cells was measured using a DAF-FMDA probe following the manufacturer’s instructions. RK-13 or NBL-6 cells were seeded into 12-well plates and treated with BR (5, 10, 20, 50, and 100 µM) for 24 h. Next, the medium was replaced with serum-free DMEM containing DAF-FMDA (5 µM/L) and incubated at 37°C for 20 min, followed by three PBS washes. The fluorescent intensity was measured by the Spark microplate reader.

### Inhibition of virus infection assays and relevant signal pathway identification

The RK-13 and NBL-6 cells were seeded into 12-well cell plates and pre-incubated with BV, FeCl_3_, CORM-3, or BR at different concentrations (0, 5, 10, 20, 50, and 100 µM). Next, the cells were infected with EqHV-8 SDLC66 at an MOI of 0.1, cells and cellular supernatant were collected to analyze EqHV-8 replication at 24 hpi by Western blotting and qPCR.

The RK-13 and NBL-6 cells were seeded into 12-well plates at a density of 1 × 10^5^ cells/well overnight and infected with EqHV-8 SDLC66 at an MOI of 0.1 for 1 h. Next, the medium was replaced with 3% FBS DMEM containing SNP and sGC-specific inhibitor ODQ (10 µM) or PKG-specific inhibitor KT5823 (2 µM). The cells and supernatant were harvested to determine the progeny virus copy number and gD protein expression by qPCR and Western blotting.

### Animal experiments

Twenty specific pathogen-free, male, 8-week-old BALB/c mice were purchased from Peng Yue Experimental Animal Breeding Co., Ltd. (Jinan, China). Next, the mice were randomly allocated to four groups (*n* = 5 mice/group) as follows: each group was housed separately to prevent cross-infection. Mice in the mock group were inoculated intranasally with 100 µL of PBS. Mice in the EqHV-8 group were inoculated intranasally with 100 µL of EqHV-8 (1 × 10^5^ PFU/mice). Mice in the CoPP group were injected intraperitoneally with 100 µL of CoPP (30 mg/kg), followed by intranasal inoculation with 100 µL of EqHV-8 (1 × 10^5^ PFU/mice), and mice in the ZnPP group were inoculated intraperitoneally with 100 µL of ZnPP (15 mg/kg) as previously described (9). This was followed by inoculating the mice intranasally with 100 µL of EqHV-8 (1 × 10^5^ PFU/mice), and CoPP or ZnPP was administered with the same dose daily after the viral infection. The body weight and clinical manifestations of all mice were monitored at 0, 2, 4, 6, and 8 dpi. Clinical manifestations were scored with a scale of marks as previously described (62). For movement, 0 indicated moving within 15 s; 1 indicated moving within 30 s, and 2 indicated no movement within 30 s. For eyes, 0 indicated bright, neither bulging, nor sunken; 1 indicated bright, eyes starting to bulge or sink; and 2 indicated dull appearance, eyes distinctly bulging or sunken. For coat, 0 indicated glossy, sleek, and even coat; 1 indicated disheveled coat, little shine; 2 indicated that coat develops bristly and is lusterless, shed hair. For posture, 0 indicated full-body stretching; 1 indicated little full-body stretching; and 2 indicated no full-body stretching. For secretions, 0 indicated no secretions around the eyes and nose; 1 indicated secretions apparent for at least one of the eyes or nose; and 2 indicated secretions apparent for both eyes and nose. Finally, these mice were euthanized via cervical dislocation at 8 dpi, and the lung tissue was collected for further histopathological analysis and detection of efficient replication.

### Histopathology and immunohistochemistry analysis

Lung samples were fixed with 10% formalin, embedded in paraffin wax, sliced in a microtome (Leica) to 4-µm sections, affixed onto slides, and then subjected to hematoxylin and eosin staining for histopathological examination and immunohistochemistry ( staining to detect the EqHV-8 antigen with positive serum as described previously (44). The slides were observed under a Leica DMi eight confocal microscope (Leica).

### Virus replicates in tissues

The EqHV-8 replication in the lungs was titrated using RK-13 cells as previously described (44). Briefly, lung tissue (0.1 g) mixed with PBS (1 mL) was crushed, homogenized, frozen, and thawed thrice. Afterward, the supernatant was collected and filtered through a 0.22-µm syringe filter and titrated using RK-13 cells according to the Reed–Muench method.

### Statistical analysis

Statistically significant differences between the groups were determined using unpaired Student’s *t*-test. Significance was indicated as follows: *, *P* < 0.05; **, *P* < 0.01; and ***, *P* < 0.001.

## SUPPLEMENTAL MATERIAL

Fig. S1 spectrum.03220-23-s0001.jpg Cell toxicity of CoPP.

Fig. S2 spectrum.03220-23-s0002.jpg Cell toxicity of ZnPP.

Fig. S3 spectrum.03220-23-s0003.jpg HO-1 expression identification.

Fig. S4 spectrum.03220-23-s0004.jpg HO-1 expression.

Fig. S5 spectrum.03220-23-s0005.jpg Cytotoxicity of RK-13 or NBL-6 cells treated with enzastaurin.

Fig. S6 spectrum.03220-23-s0006.jpg EHV-8 replication.

Fig. S7 spectrum.03220-23-s0007.jpg Cytotoxicity of RK-13 or NBL-6 cells treated with SNP.

Fig. S8 spectrum.03220-23-s0008.jpg Cytotoxicity of ODQ.

Legends spectrum.03220-23-s0009.docx Supplemental figure legends.

ASM does not own the copyrights to Supplemental Material that may be linked to, or accessed through, an article. The authors have granted ASM a non-exclusive, world-wide license to publish the Supplemental Material files. Please contact the corresponding author directly for reuse.
