# Supplementary figures and images for: Heme oxygenase-1 is an equid alphaherpesvirus 8 replication restriction host protein and suppresses viral replication via the PKCβ/ERK1/ERK2 and NO/cGMP/PKG pathway
Source: Microbiol Spectr. 2024 Mar 5;12(4):e03220-23. doi: 10.1128/spectrum.03220-23 (PMC10986571; doi:10.1128/spectrum.03220-23)

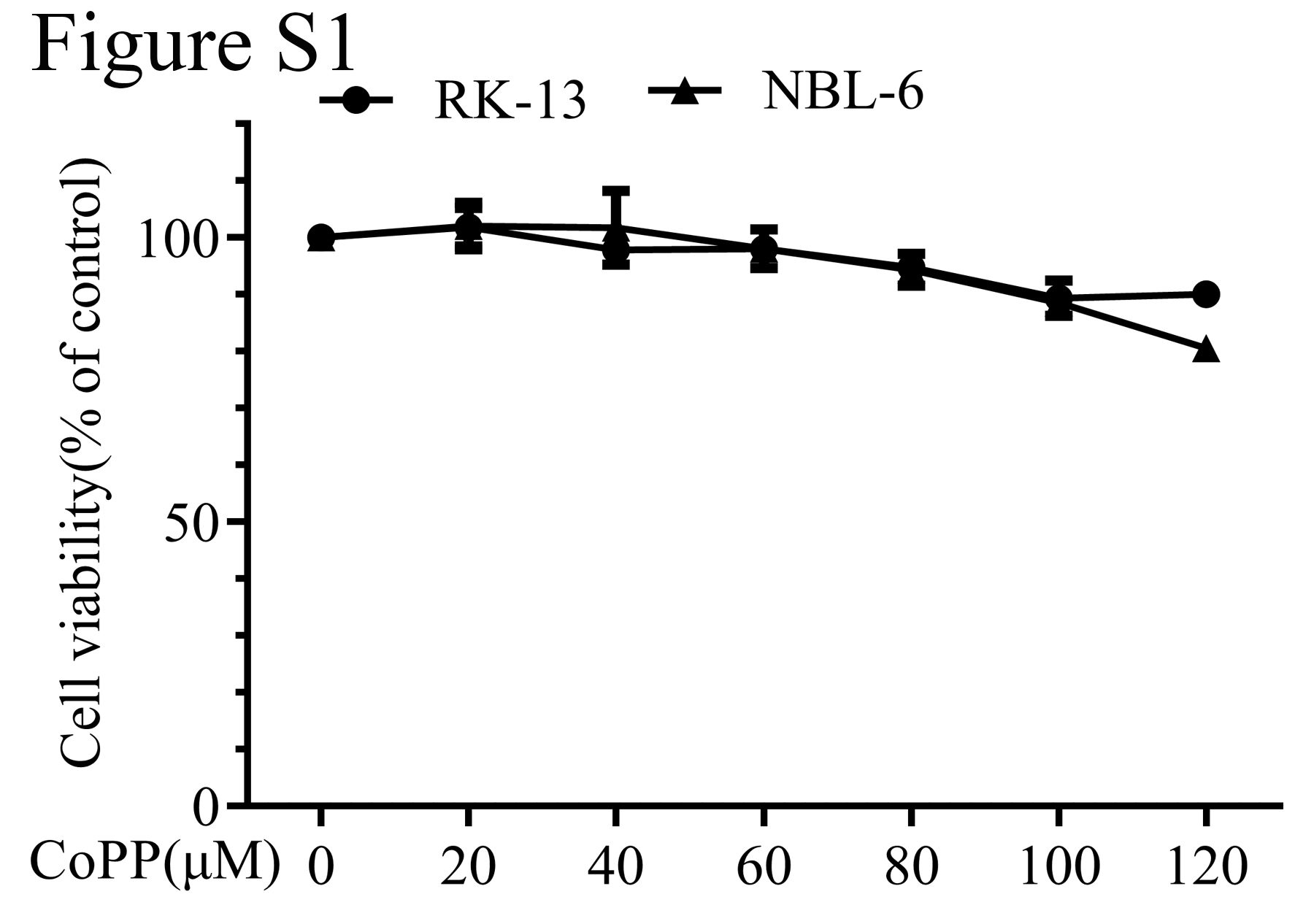

Supplement: Fig. S1 — Cell toxicity of CoPP. [file spectrum.03220-23-s0001.jpg]

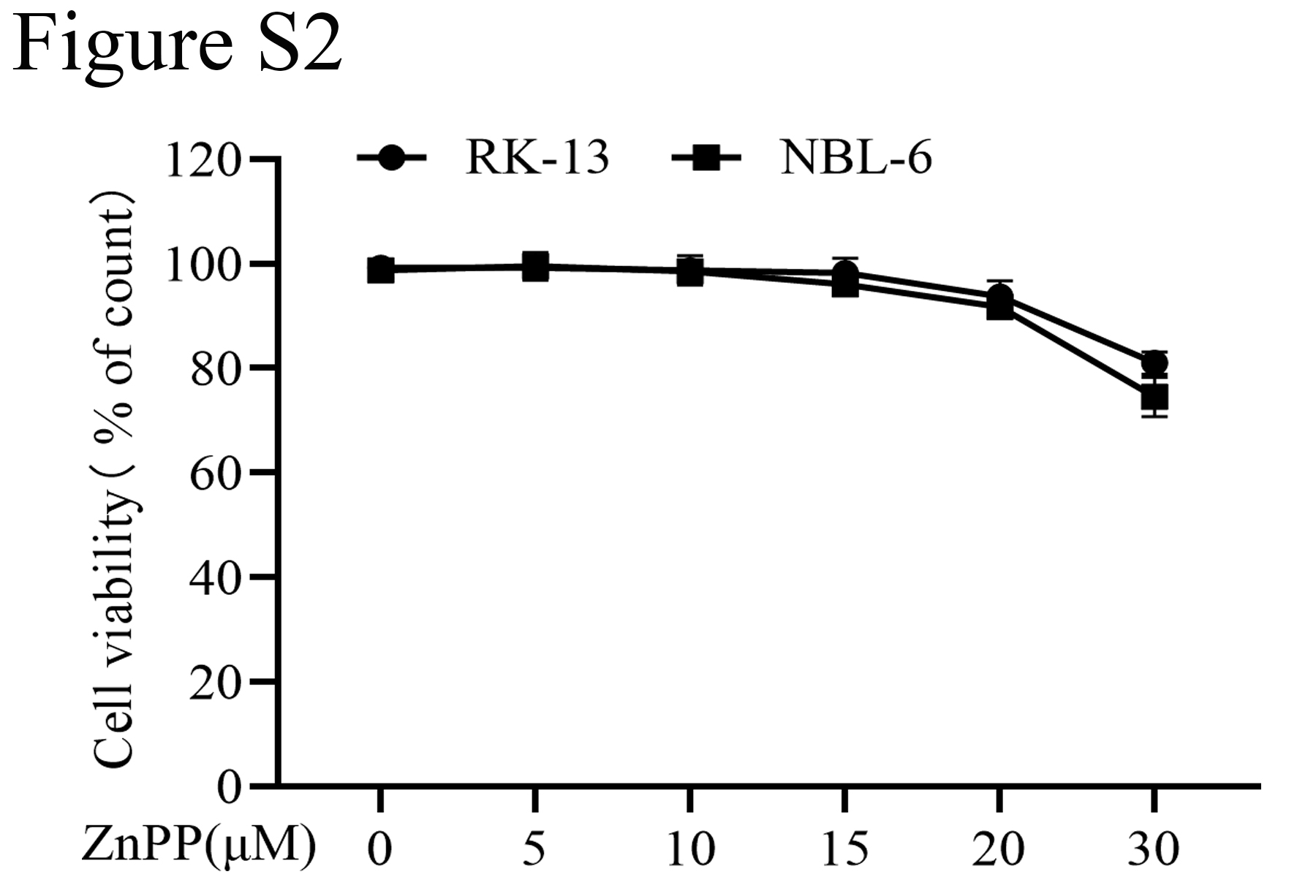

Supplement: Fig. S2 — Cell toxicity of ZnPP. [file spectrum.03220-23-s0002.jpg]

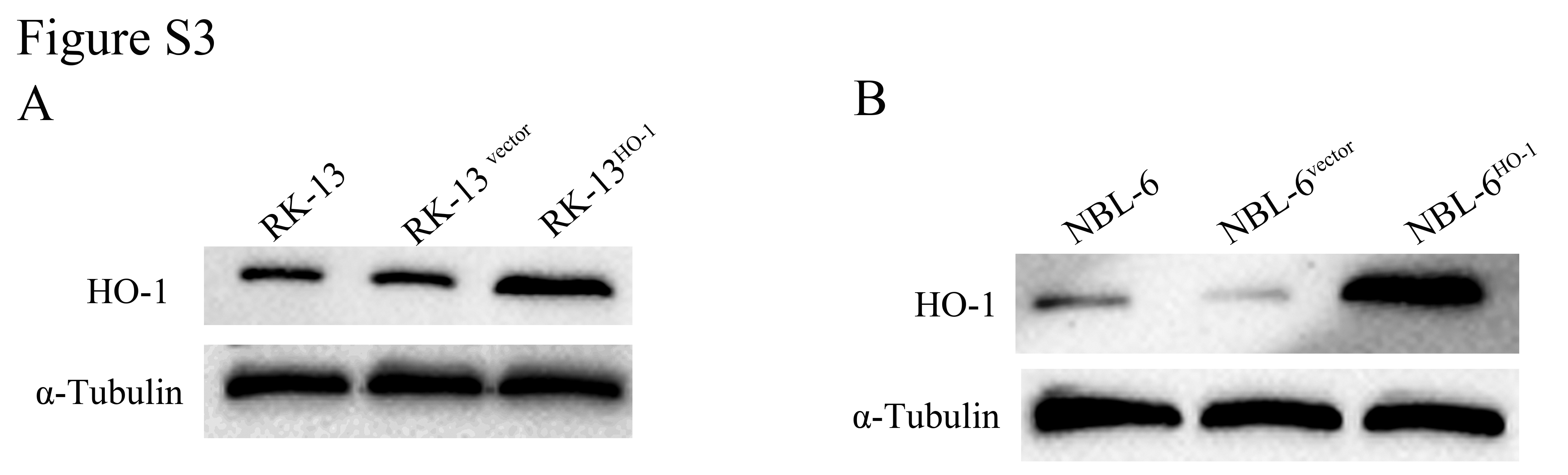

Supplement: Fig. S3 — HO-1 expression identification. [file spectrum.03220-23-s0003.jpg]

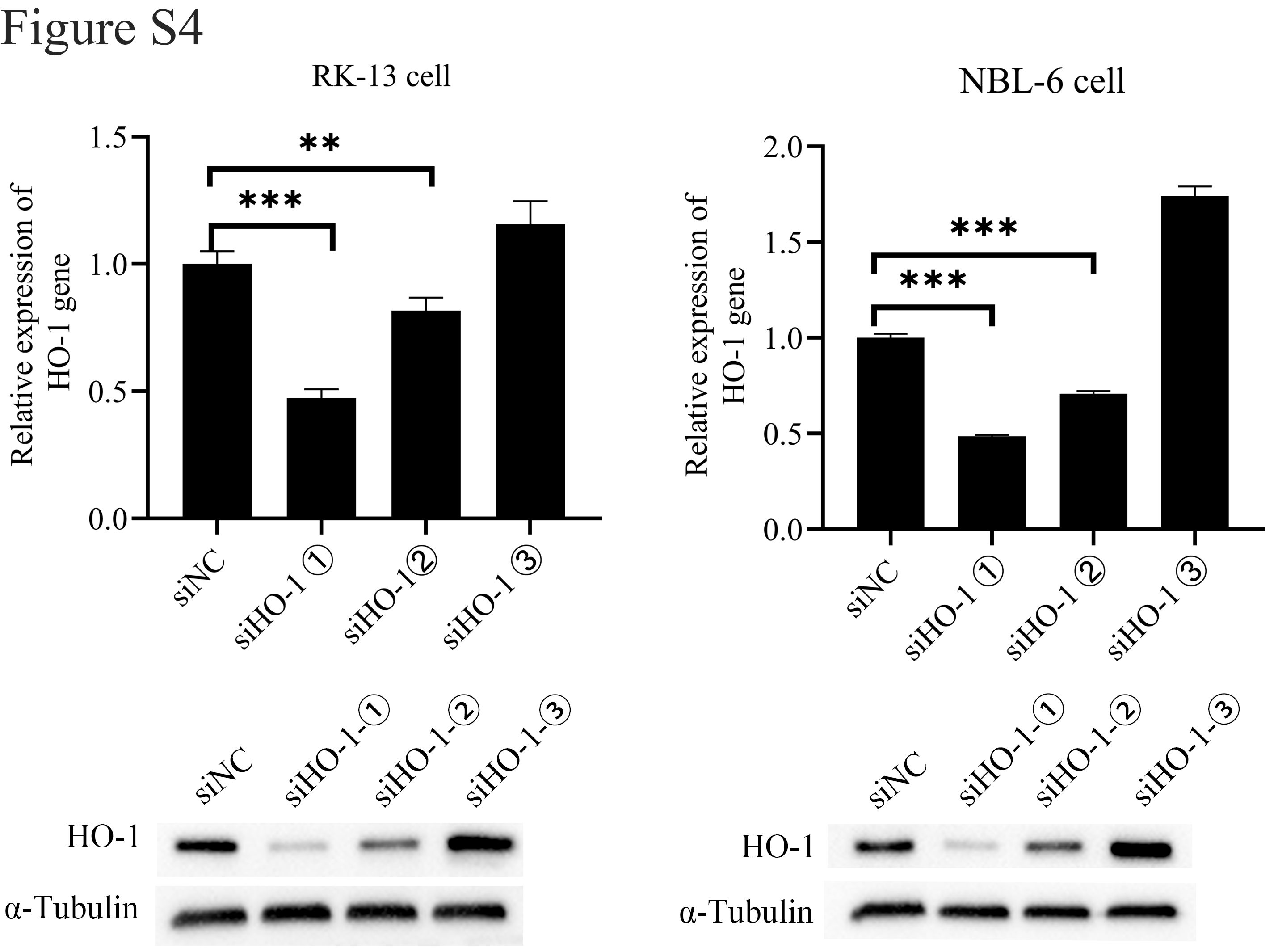

Supplement: Fig. S4 — HO-1 expression. [file spectrum.03220-23-s0004.jpg]

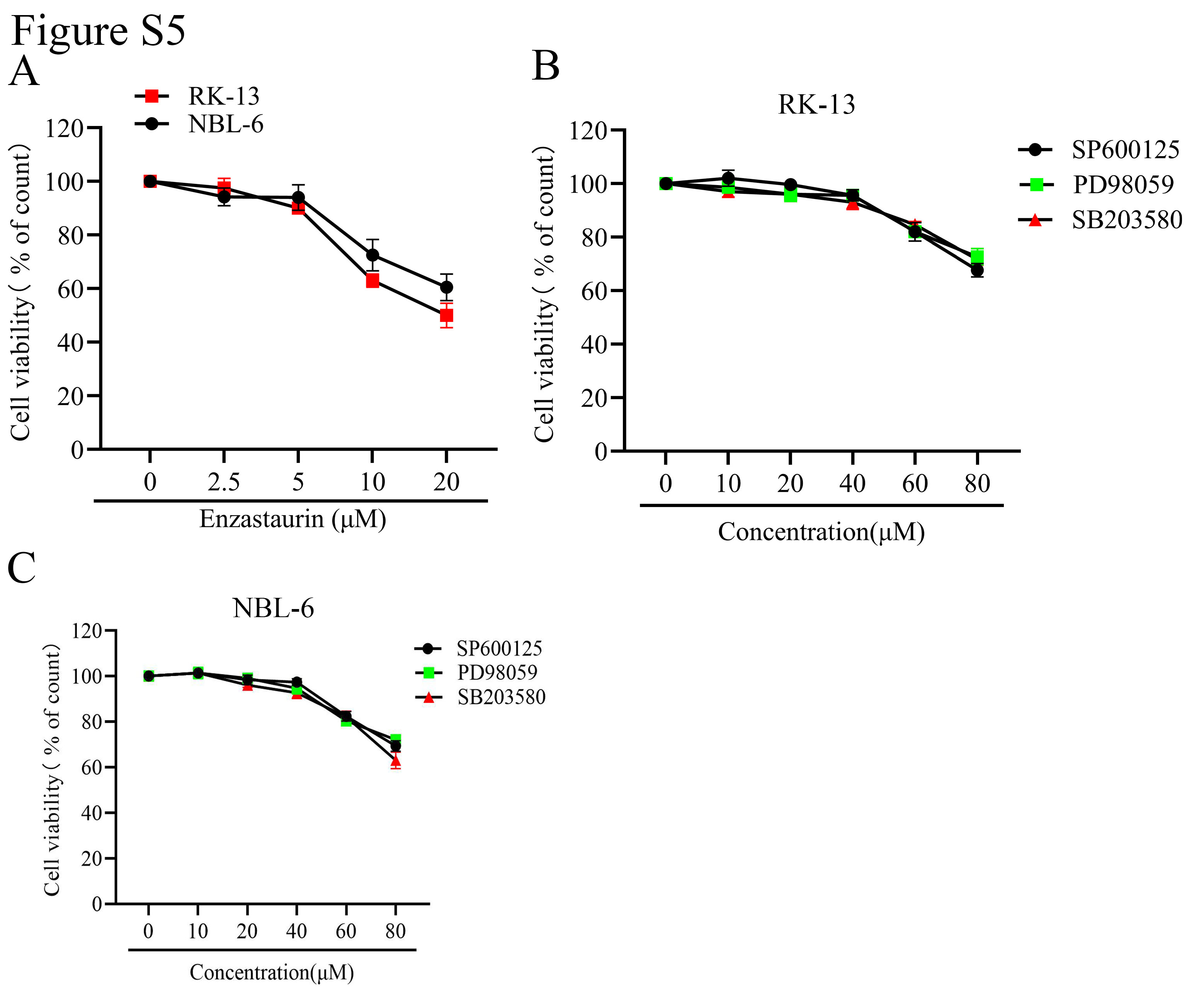

Supplement: Fig. S5 — Cytotoxicity of RK-13 or NBL-6 cells treated with enzastaurin. [file spectrum.03220-23-s0005.jpg]

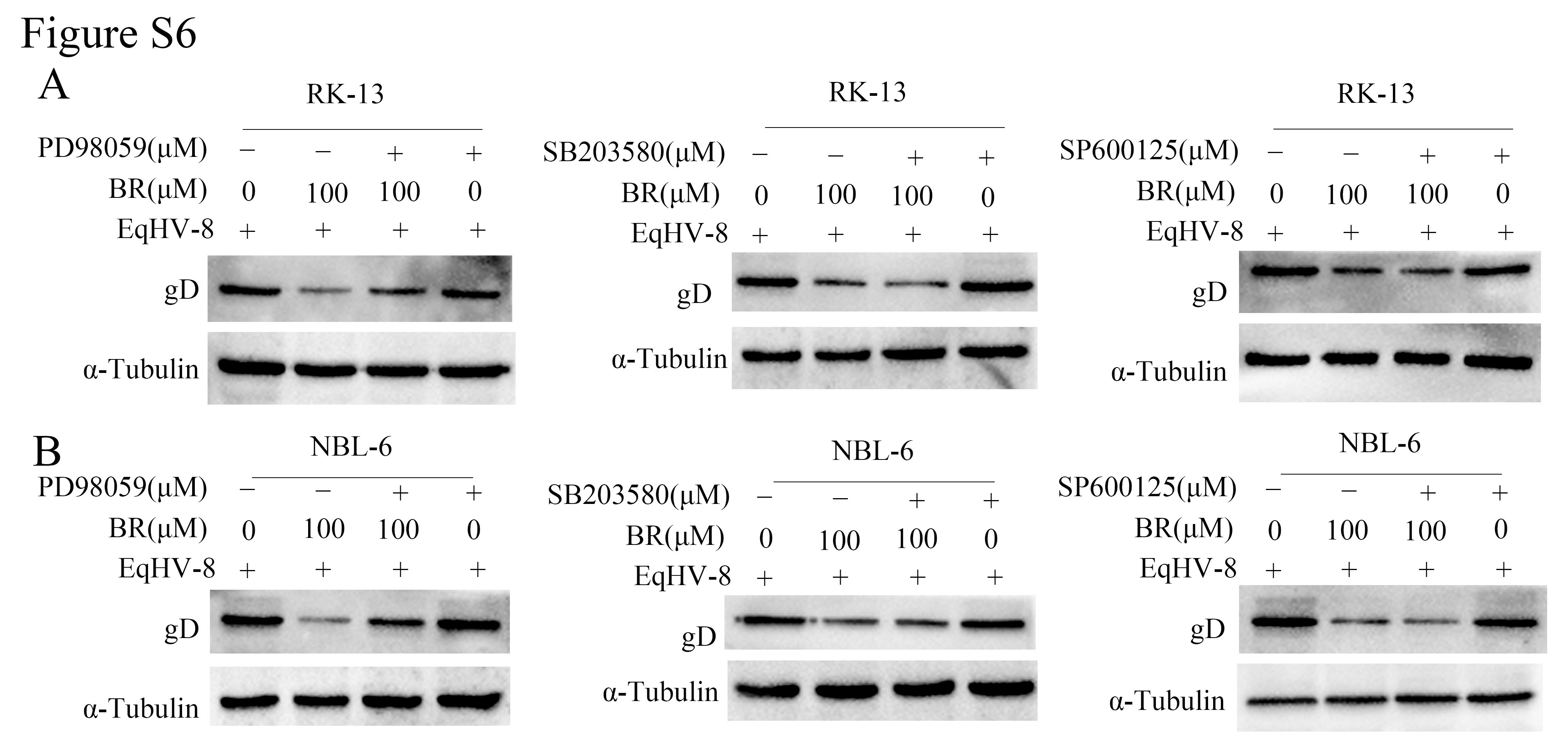

Supplement: Fig. S6 — EHV-8 replication. [file spectrum.03220-23-s0006.jpg]

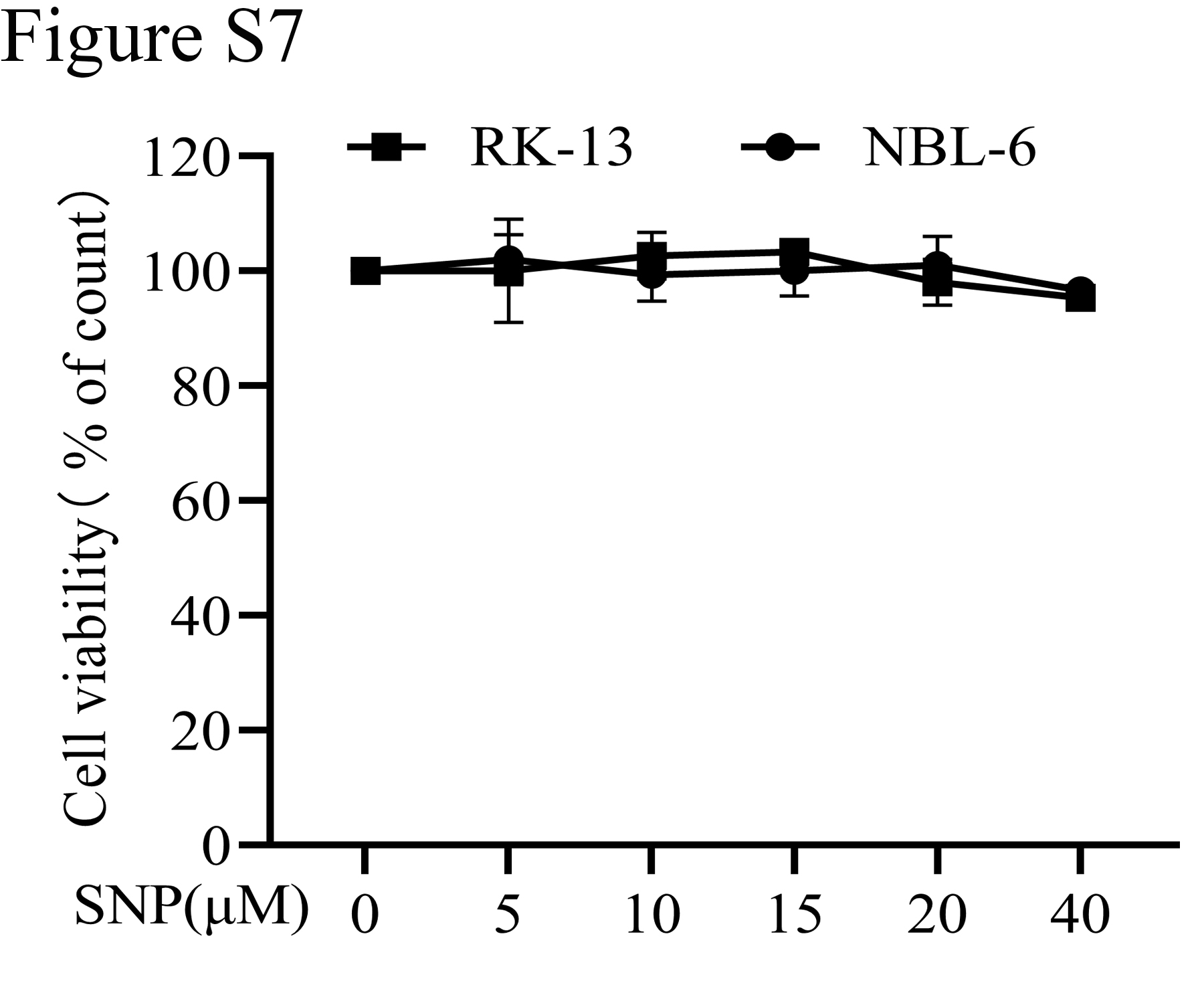

Supplement: Fig. S7 — Cytotoxicity of RK-13 or NBL-6 cells treated with SNP. [file spectrum.03220-23-s0007.jpg]

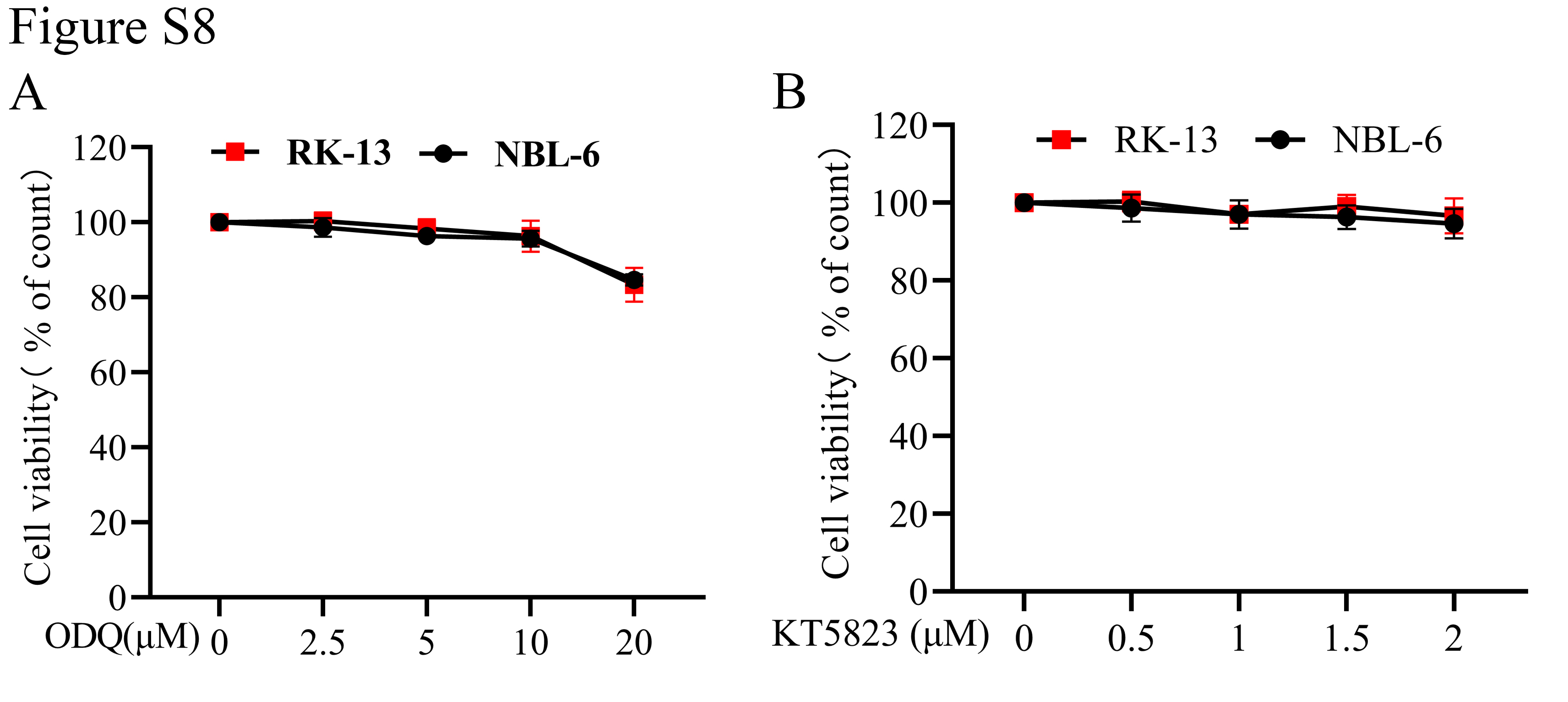

Supplement: Fig. S8 — Cytotoxicity of ODQ. [file spectrum.03220-23-s0008.jpg]
