# Supplementary material for: Heme oxygenase-1 is an equid alphaherpesvirus 8 replication restriction host protein and suppresses viral replication via the PKCβ/ERK1/ERK2 and NO/cGMP/PKG pathway
Source: Microbiol Spectr. 2024 Mar 5;12(4):e03220-23. doi: 10.1128/spectrum.03220-23 (PMC10986571; doi:10.1128/spectrum.03220-23)
Supplement: Legends — Supplemental figure legends. [file spectrum.03220-23-s0009.docx]

**Supplementary Figure legend**

**Fig. S1**

The cell toxicity of CoPP was examined in RK-13 and NBL-6 cells using CCK-8 kit and was expressed as relative cell viability by comparing with the viable cells in the absence of CoPP (set up as 100 %).

**Fig. S2**

The cell toxicity of ZnPP was examined in RK-13 and NBL-6 cells using CCK-8 and was expressed as relative cell viability by comparing with the viable cells in the absence of CoPP (set up as 100%).

**Fig. S3**

The HO-1 expression was assessed in RK-13^HO-1^, NBL-6 ^HO-1^ and their parent cells by Western blot.

**Fig. S4**

The HO-1 expression in RK-13, and NBL-6 cells with siHO-1or siNC transfection were measured by RT-PCR and Western blot. ** *p* < 0.01, *** *p* < 0.001

**Fig. S5**

The Cytotoxicity of RK-13 or NBL-6 cells treated with Enzastaurin at different concentrations (0, 2.5, 5, 10, and 20 μM) for 24 h was detected by CCK-8(A). The RK-13(B) or NBL-6 (C)were incubated with SB203580, PD98059 or SP600125 at various concentrations (10, 20, 40, 60, and 80 μM) or DMSO (represented by 0 μM SB203580, PD98059 or SP600125) for 24 h was determined by CCK-8.

**Fig. S6**

The RK-13 (A) or NBL-6 (B) cells were treated with a mixture of BV (150 µM) and PD98059 (50 µM), SB203580 (50 µM) or SP600125 (50 µM), followed by infection with EHV-8 at 0.1 MOI. EHV-8 replication was analyzed by western blotting. Data are represented as mean ± SD from three independent experiments. * *p* < 0.05, ** *p* < 0.01, *** *p* < 0.001.

**Fig. S7**

The Cytotoxicity of RK-13 or NBL-6 cells treated with SNP at different concentrations (5, 10, 15, 20, and 40 mM) or PBS (represented by 0 mM SNP) for 24 h was detected by CCK-8.

**Fig. S8**

The cytotoxicity of ODQ with different dosages (0, 2.5, 5, 10, and 20 μM) in RK-13 and NBL-6 cells were determined by CCK-8 (A). The cytotoxicity of KT5823 with various concentrations (0.5, 1, 1.5, and 2 μM) in RK-13 and NBL-6 cells were determined by CCK-8 (B).
